# Supplementary material for: Screw sense excess and reversals of helical polymers in solution
Source: Nat Commun. 2023 Mar 29;14:1742. doi: 10.1038/s41467-023-37405-z (PMC10060220; doi:10.1038/s41467-023-37405-z)
Supplement: Supplementary file 1 — Supplementary Information [file 41467_2023_37405_MOESM1_ESM.pdf]

Supplementary Information

Screw Sense Excess and Reversals of Helical Polymers in Solution

Francisco Rey-Tarrío, Rafael Rodríguez, Emilio Quiñoá and Félix Freire*

Centro Singular de Investigación en Química Biolóxica e Materiais Moleculares (CiQUS) and Departamento de Química Orgánica, Universidade de Santiago de Compostela. 15782 Santiago de Compostela (Spain) e-mail: felix.freire@usc.es

Contents

Supplementary Methods 3

- Synthesis of Monomers 4
- Synthesis of Homopolymers 10
- Synthesis of Copolymers 10
- CD and UV Irradiation Studies 10
  - Homopolymers 10
  - Low temperature studies (4 °C) 10
  - Copolymers 10
  - Irradiation Studies on a Mixture of Homopolymers 11
- Original Data to Calculate Equations 3-6 11
- GPC Studies 11

Supplementary Figures 12

Supplementary Tables 39

Supplementary References 43

Supplementary Methods

CD spectroscopy
CD measurements were done in a Jasco-720. The amount of polymer used for CD measurements was 0.3 mg/mL in the corresponding solvent.

UV-Vis spectroscopy
UV-Vis spectra were registered in a Jasco V-630. The amount of polymer used for UV measurements was 0.3 mg/mL in the corresponding solvent.

Optical rotation
Optical rotation was measured in a Jasco-P2000.

NMR spectroscopy
NMR experiments were measured in a Varian 300 operating at 300 MHz for proton NMR and 75 MHz for carbon. $CDCl_3$ signal (δ= 77.2 ppm) or DMSO-d_6 (δ= 39.5 ppm) was used as standard for ^1H or ^{13}C experiments.

Optical rotation
Optical rotation was measured in a Jasco P-200

GPC Studies
GPC studies were carried out in a Waters Alliance equipped with Phenomenex GPC columns. The amount of polymer used for GPC measurements was 0.5 mg/mL in THF.

Synthesis of monomers

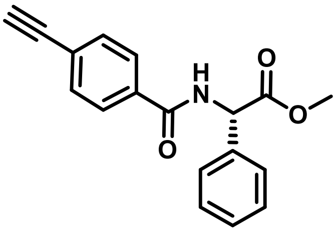<br>mono-(*S*)-**1**	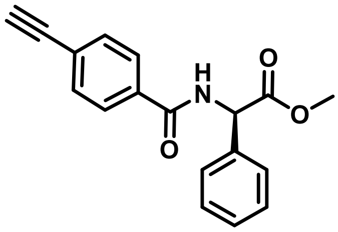<br>mono-(*R*)-**1**
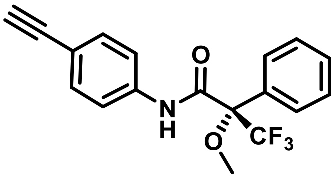<br>mono-(*S*)-**2**	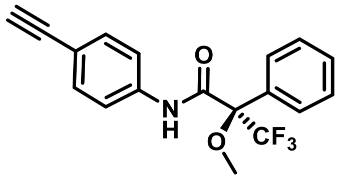<br>mono-(*R*)-**2**
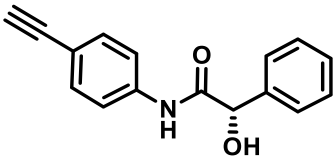<br>mono-(*S*)-**3**	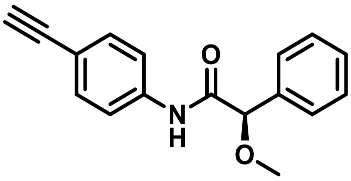<br>mono-(*R*)-**4**
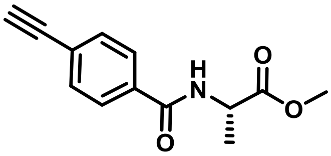<br>mono-(*S*)-**5**	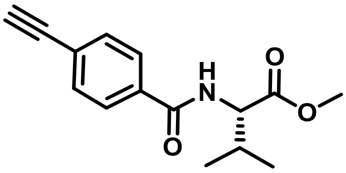<br>mono-(*S*)-**6**
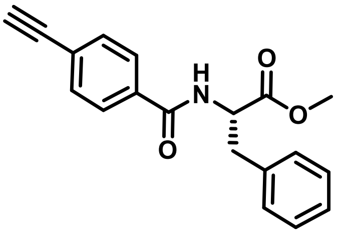<br>mono-(*S*)-**7**	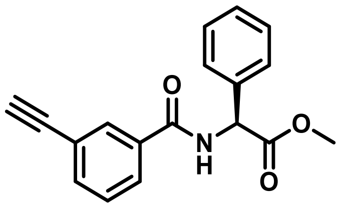<br>mono-(*S*)-**8**
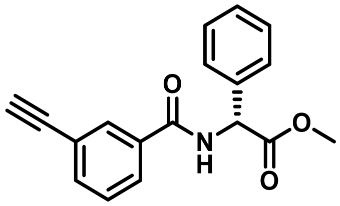<br>mono-(*R*)-**8**	

Supplementary Figure 1. Structure of the monomers synthesized.

Mono-1

1-Ethyl-3-(3-dimethylaminopropyl)carbodiimide (EDC, 1.35 g, 1.2 equiv), hydroxybenzotriazole (HOBt, 1.4 g, 1.2 equiv), (1.7 g, 1.2 equiv), 4-ethynylbenzoic acid and diisopropyltriethylamine (DIEA, 1 ml, 2 equiv) were dissolved in 70 mL of CH_2Cl_2, and the mixture was stirred for 15 min to activate the acid. Then, (*S*)-phenylglycine methyl ester or (*R*)-phenylglycine methyl ester (1.15 g, 1.0 equiv) were added and the reaction mixture was stirred overnight. The organic layer was washed with HCl 1M, saturated solution of $NaHCO_3$ and brine. The combined organic layers were dried over anhyd Na_2SO_4, filtered and the solvent was evaporated at reduced pressure. The crude product was chromatographed on silica gel (70-230 mesh) with hexane/ethyl acetate (7/3) as eluent [1.32 g Mono-(*S*)-**1**; 1.39 g mono-(*R*)-**1**], 75% and 76% yield of pure products, respectively.

^{1}H NMR (250 MHz, $CDCl_3$) δ(ppm): 3.21 (s, 1H), 3.76 (s, 3H), 5.75 (d, J = 6.9 Hz, 1H), 6.22 (d, J = 6.4 Hz, 1H), 7.34-7.45 (m, 5H), 7.53 (d, J = 8.2 Hz, 2H), 7.77 (d, J = 8.3 Hz, 2H).

^{13}C NMR (62.5 MHz, $CDCl_3$) δ(ppm): 52.9, 58.8, 79.7, 82.6, 125.7, 127.1, 127.3, 128.6, 129.0, 132.2, 133.4, 136.3, 165.7, 171.4.

$[\alpha]_D$ = -66 (c = 1.1, $CHCl_3$).

HRMS (ESI) m/z calcd for $C_{18}H_{16}NO_3$ [M + H]: 294.1125, found: 294.1121

Ref. *Angew. Chem. Int. Ed.* 2010, 49, 1430-1433.

Mono-2

Oxalyl chloride (2.5 mL, 1.4 equiv) was added dropwise to a solution of (*R*)- or (*S*)- α-methoxy- α-(trifluoromethyl)phenylacetic acid (1.00 g, 1.0 equiv) in dry hexane and DMF (300 "L, 1.0 equiv) at 0 ºC under an Ar atm. After 4 h stirring at rt, the reaction mixture was filtered, and the solution obtained dried under vacuum to give the acetyl chloride as colorless oil in quantitative yield. The (*S*)- or (*R*)- α-methoxy-α-(trifluoromethyl)phenylacetyl chloride obtained, (1.07 g, 1.0 equiv) was dissolved in CH2Cl2 (40.0 mL) and diisopropyltriethylamine (DIEA, 1.2 mL, 1.6 equiv) and 4-ethynilaniline (500.0 mg, 1.0 equiv) where added to the solution. The reaction mixture was stirred at rt overnight. The residue was diluted with CH_2Cl_2 and the organic solution was washed with HCl 1M, saturated $Na(HCO_3)$ aq solution and saturated NaCl aq solution; then the organic layer was dried over anhyd Na_2SO_4. After filtration, the solution was evaporated and the residue was chromatographed on silica gel with hexane-AcOEt (7/3, v/v) as the eluent [1.30 g mono-(*R*)-**2** and 1.28 g, mono-(*S*)-**2**], 91% and 90 % yield respectively of pure products).

Mono-(*R*)-**2**:

^{1}H NMR (300 MHz, $CHCl_3$) δ(ppm): 3.04 (s, 1H), 3.47 (s, 3H), 7.40-7.50 (m, 5H), 7.52-7.57 (m, 4H), 8.61 (broad s, 1H)

19F NMR (282.3 MHz, $CHCl_3$) δ (ppm): -68.7

^{13}C NMR (100 MHz, $CHCl_3$) δ (ppm): 55.2, 83.1, 118.5, 119.5, 127.7, 128.0, 128.8, 129.0, 129.8, 132.0, 133.0, 137.1, 164.3.

HRMS (ESI) m/z calcd for $C_{18}H_{15}F_3NO_2$ [M+H]: 334.1010, found: 334.1039.

$[\alpha]_D$= +68.2 (c = 10.0 mg/mL, $CHCl_3$).

Mono-(*S*)-**2**

^1H NMR (300 MHz, $CHCl_3$) δ(ppm): 3.04 (s, 1H), 3.47 (s, 3H), 7.40-7.50 (m, 5H), 7.52-7.57 (m, 4H), 8.61 (broad s, 1H).

^{19}F NMR (282.3 MHz, $CHCl_3$) δ (ppm): -68.7.

^{13}C NMR (100 MHz, $CHCl_3$) δ (ppm): 55.2, 83.1, 118.5, 119.5, 127.7, 128.0, 128.8, 129.0, 129.8, 132.0, 133.0, 137.1, 164.3.

HRMS (ESI) m/z calcd for $C_{18}H_{15}F_3NO_2$ [M + H]: 334.1010, found: 334.1049.

$[\alpha]_D$ = -67.7 (c = 10.0 mg/mL, $CHCl_3$).

Ref. *Chem. Sci.* 2013, 4, 2735-2743.

Mono-(*S*)-3

Mono-**3** in prepared trough a four-step protocol:

1st Step

TMSCl and (S)-α-hidroxy-α-phenylacetic acid (1 g, 6.6 mmol, 1 equiv.) were dissolved in 35 mL of dichloromethane, pyridine (Py, 1.3 mL, 16.4 mmol, 2.5 equiv) and DMAP (0.04 g, 0.33 mmol, 0.05 equiv) were added and the reaction was stirred for 15 min to activate. Then, chlorotrimethylsilane (TMSCl, 2mL, 16.5 mmol, 2.5 equiv) was added and the reaction mixture was stirred during 4 hours at room temperature.

2nd Step

After 4 hours, a drop of DMF dissolved in 1 mL of both dichloromethane and oxalylchloride were added to the reaction. The reaction was stirred at 0ºC for 1 hour.

3rd Step

4-ethynylaniline (0.77 g, 0.66 mmol, 1 equiv) dissolved in 1 mL of Pyridine was added to the reaction drop by drop at 0ºC and then the reaction was stirred overnight at room temperature.

4th Step

Citric acid (1.26 g, 0.66 mmol, 1 equiv) dissolved in 15 mL of methanol was added and the reaction was stirred for 8 hours. Then, the organic layer was washed with HCl 1M, saturated solution of $NaHCO_3$ and brine. The combined organic layers were dried over anhyd. Na_2SO_4, filtered and the solvent was evaporated at reduced pressure. The crude product was chromatographed on silica gel (70-230 mesh) with hexane/ethyl acetate (8/2) as eluent [1.24 g mono-**3**], 75% yield of pure product.

^1H NMR (300 MHz, $CDCl_3$) δ(ppm): 2.95 (s, 1H), 4.23 (s, 3H), 4.85 (s, 1H), 7.15-7.30 (m, 9H), 8.51 (s, 1H).

^{13}C NMR (62.5 MHz, $CDCl_3$) δ (ppm): 74.5, 83.3, 118.3, 119.7, 126.8, 128.8, 133.9, 137.3, 138.9, 170.8.

HRMS (ESI) m/z calcd for $C_{17}H_{15}NO_2$ [M+Na]: 274.0829, found: 274.0838.

$[\alpha]_D$= +43.7 (c= 20 mg/mL, $CHCl_3$).

Ref. *Macromolecules*, 2020, 53, 3182-3193.

Mono-(*R*)-4

(2-(7-Aza-1H-benzotriazole-1-yl)-1,1,3,3-tetramethyluronium hexafluorophosphate), 2.30 g, 1.4 equiv), 1-hydroxybenzotriazole (HOBt, 0.160 mg, 1.4 equiv), (*R*)-α-methoxy-α-phenylacetic acid (850 mg, 1.4 equiv) and diisopropyltriethylamine (207 µL, 1.4 equiv) were dissolved in 45 mL of CH_2Cl_2, and the mixture was stirred for 10 min to activate the acid. Then, 4-ethynylaniline (0.500 g, 1.0 equiv) was added and the reaction mixture was stirred for 1 day. Then, the organic layer was washed with water, HCl 1M, water and a saturated solution of $NaHCO_3$. The combined organic layers were dried over anhydrous Na_2SO_4, filtered and the solvent was evaporated at reduced pressure. The crude product was chromatographed on silica gel (70-230 mesh) with hexane/ethyl acetate (7/3) as eluent (0.980 g, 89% yield).

^{1}H NMR (250 MHz, MeCN) δ(ppm): 3.39 (s, 1H), 3.40 (s, 3H), 4.75 (s, 1H), 7.34-7.46 (m, 7H), 7.65-7.67 (m, 2H), 9.01 (broad s, 1H).

^{13}C NMR (62.5 MHz, MeCN) δ(ppm): 57.7, 78.3, 83.9, 84.6, 128.1, 129.3, 129.4, 133.4, 138.3, 139.5, 170.1.

$[\alpha]_D$= +15 (c= 1.1 mg/mL, $CHCl_3$).

HRMS (ESI) m/z calcd for $C_{18}H_{16}NO_3$ [M + H]: 265.3065, found: 265.2948.

Ref. *Angew. Chem. Int. Ed.* 2011, 50, 11692-11696.

Mono-(*S*)-5

1-Ethyl-3-(3-dimethylaminopropyl)carbodiimide (EDC, 1.350 g, 1.2 equiv.), hydroxybenzotriazole (HOBt, 1.400 g, 1.2 equiv.), 2-ethynylbenzoic acid (1.700 g, 1.2 equiv) and diisopropyltriethylamine (DIPEA, 1 mL, 2.0 equiv.) were dissolved in 70 mL of CH2Cl2, and the mixture was stirred for 15 min to activate the acid. Then, methyl L-alaninate (1.151 g, 1.0 equiv) was added and the reaction mixture was stirred overnight. The organic layer was washed with HCl 1M, saturated solution of NaHCO3 and brine. The combined organic layers were dried over anhyd Na_2SO_4, filtered and the solvent was evaporated at reduced pressure. The crude product was chromatographed on silica gel (70-230 mesh) with hexane/ethyl acetate (7/3) as eluent [2.112 g Mono-(*S*)-**5**], 82% of pure product.

^{1}H NMR (300 MHz, $CDCl_3$) δ (ppm): 1.49 (d, 3H), 3.25 (s, 1H), 3.74 (s, 3H), 4.75 (q, 1H), 7.29 (d, 1H), 7.48 (d, 2H), 7.76 (d, 2H).

^{13}C NMR (75 MHz, $CDCl_3$) δ (ppm): 18.0, 48.5, 52.4, 79.7, 82.7, 125.4, 127.1, 132.0, 133.6, 166.2, 173.5.

$[\alpha]_D$= +81 (15 mg mL^{-1} , $CHCl_3$)

Ref. *Polym. Chem.*, 2017,8, 3740-3745

Mono-(*S*)-6

1-Ethyl-3-(3-dimethylaminopropyl)carbodiimide (EDC, 0.656, 1.2 equiv.), hydroxybenzotriazole (HOBt, 0.465 g, 1.2 equiv), 2-ethynylbenzoic acid (0.500 g, 1.2 equiv) and diisopropyltriethylamine (DIPEA, 695 mL, 2.0 equiv) were dissolved in 50 mL of CH2Cl2, and the mixture was stirred for 15 min to activate the acid. Then, methyl *L*-valinate (0.398 g, 1.0 equiv) was added and the reaction mixture was stirred overnight. The organic layer was washed with HCl 1M, saturated solution of $NaHCO_3$ and brine. The combined organic layers were dried over anhyd Na_2SO_4, filtered and the solvent was evaporated at reduced pressure. The crude product was chromatographed on silica gel (70-230 mesh) with hexane/ethyl acetate (7/3) as eluent [0.597 g Mono-(*S*)-**6**], 75% of pure product.

^{1}H NMR (300 MHz, $CDCl_3$) δ (ppm): 0.96 (m, 6H), 2.24 (m, 1H), 3.19 (1H, s), 3.73 (3H, s), 4.72 (q, 1H), 6.73 (d, 1H), 7.50 (d, 2H), 7.73 (d, 2H).

^{13}C NMR (75 MHz, CDCl3) δ (ppm): 18.1, 19.0, 31.6, 52.3, 71.3, 79.7, 82.8, 125.6, 127.1, 132.3, 134.1, 166.5, 172.6

$[\alpha]_D$= +36 (15 mg mL^{-1}, $CHCl_3$)

Ref. *Polym. Chem.*, 2017,8, 3740-3745

Mono-(*S*)-7

1-Ethyl-3-(3-dimethylaminopropyl) carbodiimide (EDC, 0.656 g, 1.2 equiv), hydroxybenzotriazole (HOBt, 0.465 g, 1.2 equiv), 2-ethynylbenzoic acid (0.500 g, 1.2 equiv) and diisopropyltriethylamine (DIPEA, 0.70 mL, 2.0 equiv) were dissolved in 50 mL of CH2Cl2, and the mixture was stirred for 15 min to activate the acid. Then, methyl *L*-phenylalaninate, (0.500 g, 1.0 equiv) was added and the reaction mixture was stirred overnight. The organic layer was washed with HCl 1M, saturated solution of $NaHCO_3$ and brine. The combined organic layers were dried over anhyd Na_2SO_4, filtered and the solvent was evaporated at reduced pressure. The crude product was chromatographed on silica gel (70-230 mesh) with hexane/ethyl acetate (7/3) as eluent. After purification, 0.690 g (83%) of Mono-(*S*)-**7** were obtained.

^1H NMR (300 MHz, $CDCl_3$) δ (ppm): 3.19 (s, 1H), 3.25 (m, 2H), 3.77 (s,1H), 5.08 (q, 1H), 6.56 (d, 1H), 7.12 (m, 2H), 7.28 (m, 3H), 7.53 (d, 2H), 7.68 (d, 2H).

^{13}C NMR (75 MHz, $CDCl_3$) δ (ppm): 17.4, 52.3, 53.7, 79.8, 82.6, 125.5, 127.0, 127.1, 128.5, 129.1, 132.1, 133.6, 135.9, 166.2, 172.0.

$[\alpha]_D$= +51 (15.0 mg/mL, $CHCl_3$)

Ref. *Chem Commun*, 2017, *53*, 8573-8576

Mono-(*R*)-8 and Mono-(*S*)-8

(1-Ethyl-3-(3-dimethylaminopropyl)carbodiimide) (EDC, 1.35 g, 1.2 equiv.), hydroxibenzotriazole (HOBt, 1.4 g, 1.2 equiv.), (1.7 g, 1.2 equiv.), 3-ethynylbenzoic acid and diisopropyltriethylamine (DIEA, 1 ml, 2 equiv.) were dissolved in 70 mL of CH_2Cl_2, and the mixture was stirred for 15 min to activate the acid. Then, (R)- or (S)-phenylglycine methylester (1.15 g, 1.0 equiv.) was added and the reaction mixture was stirred overnigth. The organic layer was washed with HCl 1M, saturated solution of $NaHCO_3$ and Brine. The combined organic layers were dried over anhydrous Na_2SO_4, filtered and the solvent was evaporated at reduced pressure. The crude product was chromatographed on silica gel (70-230 mesh) with hexane/ethyl acetate (7/3) as eluent [1.32 g Mono-(*R)*-**8** and 1.34 1.32 g Mono-(*S)*-**8**], 75% and 76% yield respectively of pure products.

(R)

α_D= +71 (c= 5, $CHCl_3$).

^1H NMR (300 MHz, $CHCl_3$) δ(ppm): 3.13 (s, 1H), 3.78 (s, 3H), 5.77 (s, J= 7.0 Hz, 1H), 7.11 (m, J= 7.0 Hz, 1H), 7.32-7.45 (m, 6H), 7.61-7.64 (m, 1H), 7.79-7.83 (m, 1H), 7,92 (s, 1H).

^{13}C NMR (62.5 MHz, $CHCl_3$) δ(ppm): 57.7, 78.3, 83.9, 84.6, 128.1, 129.3, 129.4, 133.4, 138.3, 139.5, 170.1.

HRMS (ESI) m/z calcd for $C_{18}H_{15}NO_3Na$ $[M + Na]^+$: found: 316.0946

(S)

α_D= -71 (c= 5, $CHCl_3$).

^1H NMR (250 MHz, $CHCl_3$) δ(ppm): 3.39 (s, 1H), 3.40 (s, 3H), 4.75 (s, 1H), 7.34-7.46 (m, 7H), 7.65-7.67 (m, 2H), 9.01 (broad s, 1H).

^{13}C NMR (62.5 MHz, $CHCl_3$) δ(ppm): 57.7, 78.3, 83.9, 84.6, 128.1, 129.3, 129.4, 133.4, 138.3, 139.5, 170.1.

HRMS (ESI) m/z calcd for $C_{18}H_{15}NO_3Na$ $[M + Na]^+$:, found: 316.0944

Ref. *J. Am. Chem. Soc.* 2016, 9620-9628.

Synthesis of homopolymers

Homopolymers were prepared following the general protocol for polymerization that can be found in the Methods Section of the manuscript.
See Supplementary Table 1.
See Supplementary Figures 2-9.

Synthesis of copolymers

Copolymers were prepared following the general protocol for polymerization that can be found in the Methods Section of the manuscript.
See Supplementary Tables 2-4.
See Supplementary Figures 10-22.

CD and UV irradiation studies

Homopolymers

See Supplementary Figures 23-32.

Low temperature studies (4 °C)

Low temperature studies were performed introducing the light source (Asahi Spectra Xenon light model MAX-303) and solutions in a cold room stabilized at 4 °C. After 30 minutes to stabilize the temperature of the introduced material, irradiation experiments were carried out following the general protocol for PPA irradiation.
Poly-(*R*)-**1.**
See Supplementary Figures 33-40.

Copolymers

See Supplementary Figures 41-57.

Irradiation studies on a mixture of homopolymers

Random copolymerization results in increased PEC rates due to the presence of reversals in the helix. On the other hand, if we perform studies on a mixture of poly-(R)-**1** and poly-(S)-**1**, no changes are observed in the time taken to reach a null ECD vinyl signal. Furthermore, due to the parallel decay in both helices, P and M, the CD remains unchanged until one helical sense is consumed in the case of presenting a single helix as seen for poly-(R)-**1**.

See Supplementary Figure 58.

Original data to calculate equations 3-6

See Supplementary Table 5.

GPC Studies

GPC data were obtained in an Alliance 2695 HPLC with a UV-2489 detector (Waters). The samples were eluted by three Phenogel columns connected to each other with stationary phases of 103, 104 and 105 Armstrong and packed with a solid support of a cross-linked styrene and p-divinylbenzene copolymer. GPC data for the polymers in this manuscript can be found in the tables below.

See Supplementary Tables 6-9.

Supplementary Figures

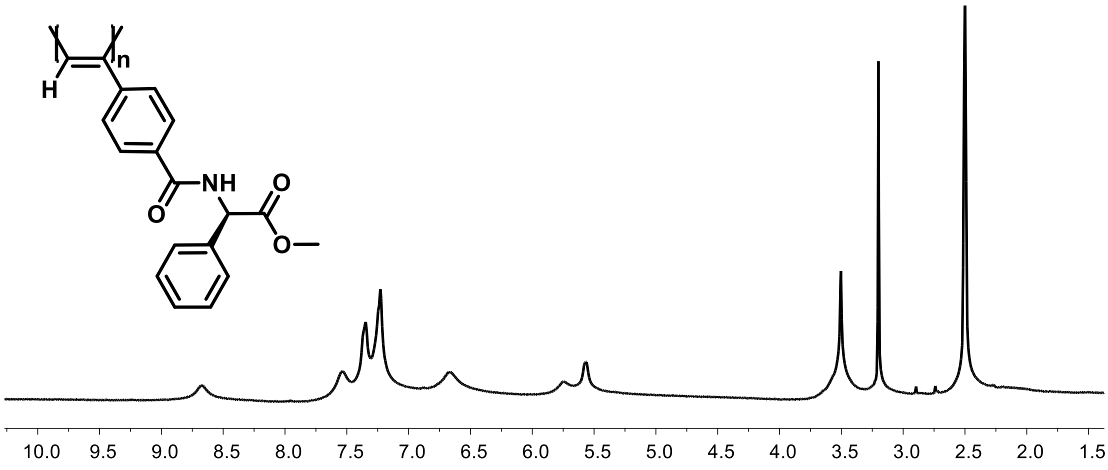

Supplementary Figure 2. ^{1}H NMR spectra of poly-(*R*)-**1** (DMSO, 300 MHz).

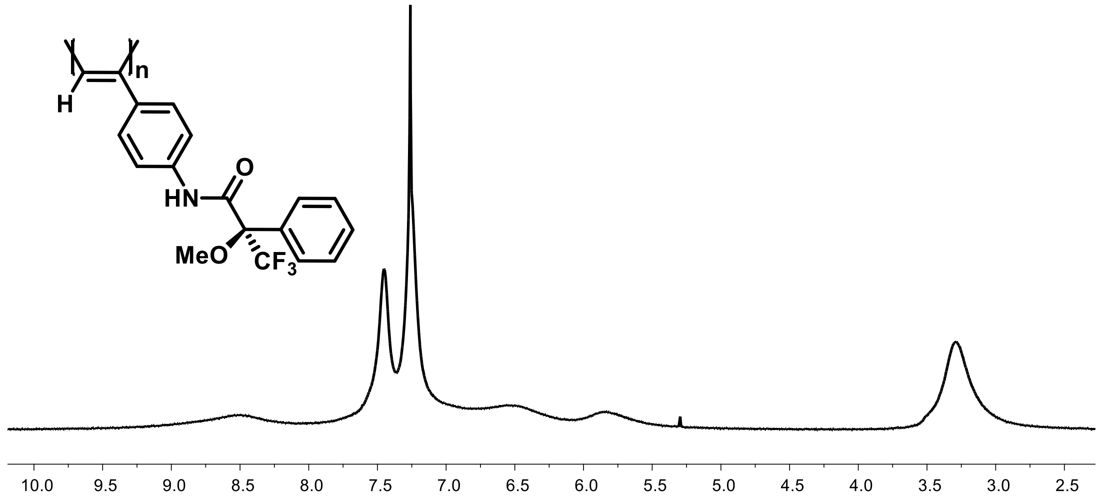

Supplementary Figure 3. ^{1}H NMR spectra of poly-(*R*)-**2** ($CDCl_3$, 300 MHz).

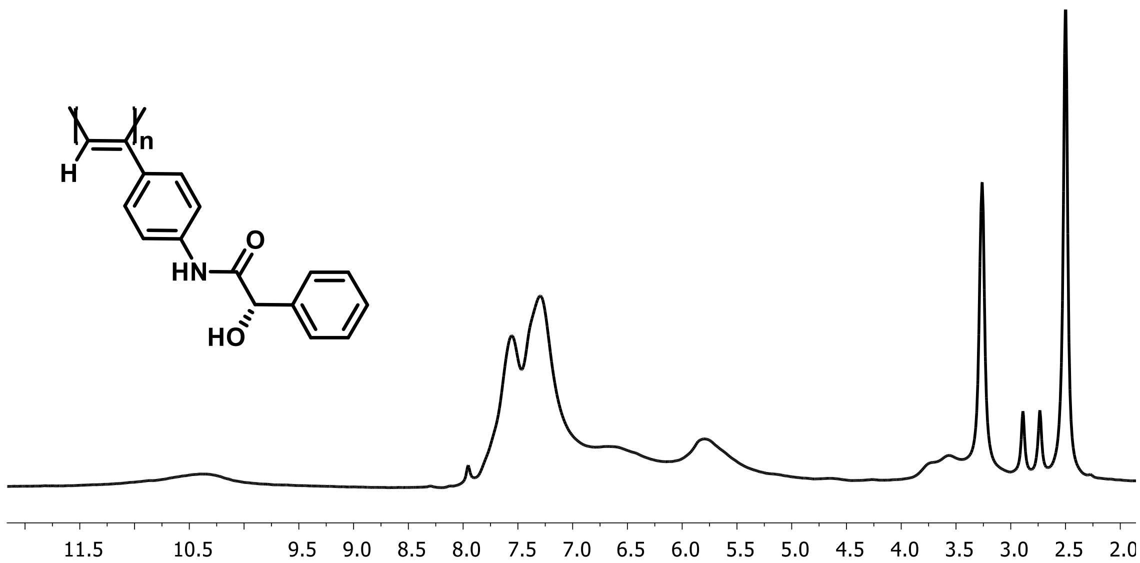

Supplementary Figure 4. ^{1}H NMR spectra of poly-(*S*)-**3** (DMSO, 300 Mhz).

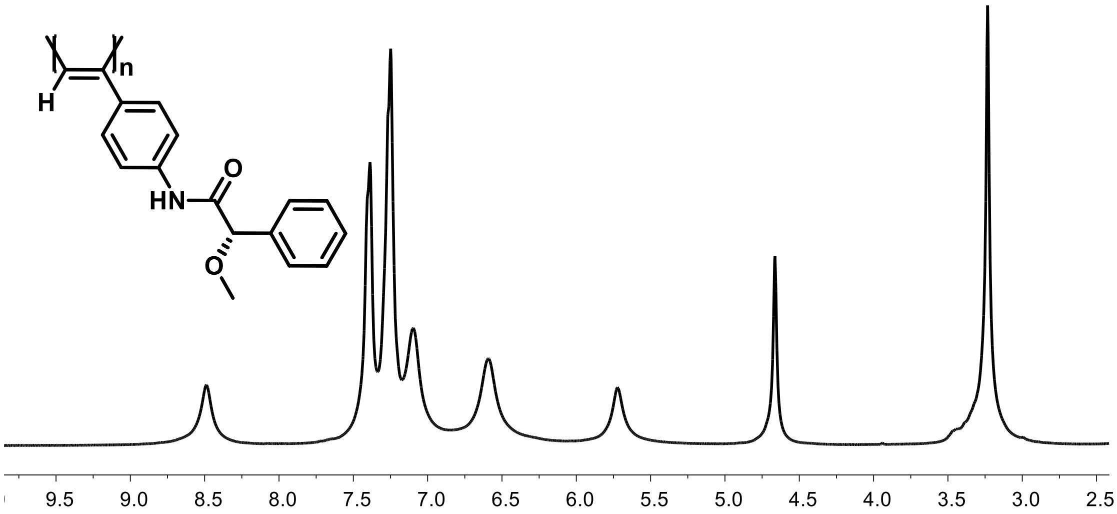

Supplementary Figure 5. ^{1}H NMR spectra of poly-(*R*)-**4** ($CDCl_3$, 300 MHz).

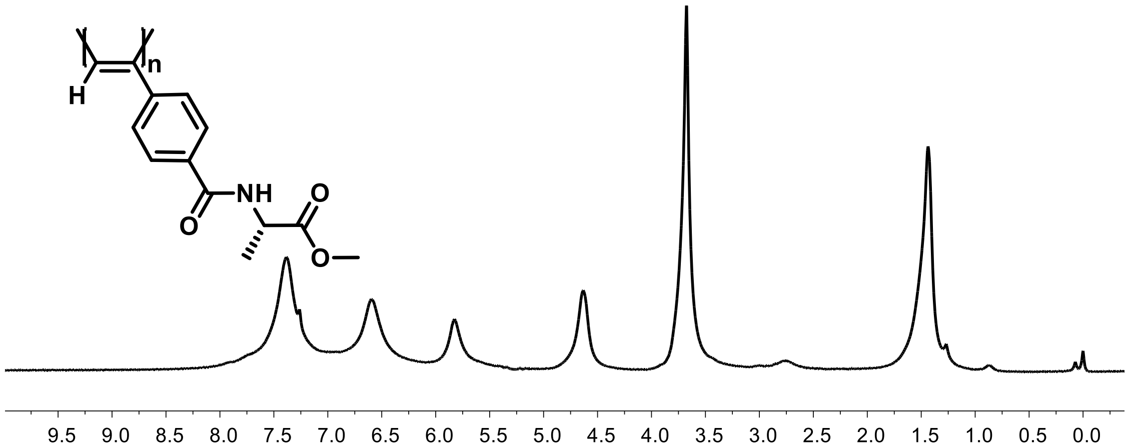

Supplementary Figure 6. ^{1}H NMR spectra of poly-(*S*)-**5** ($CDCl_3$, 300 MHz).

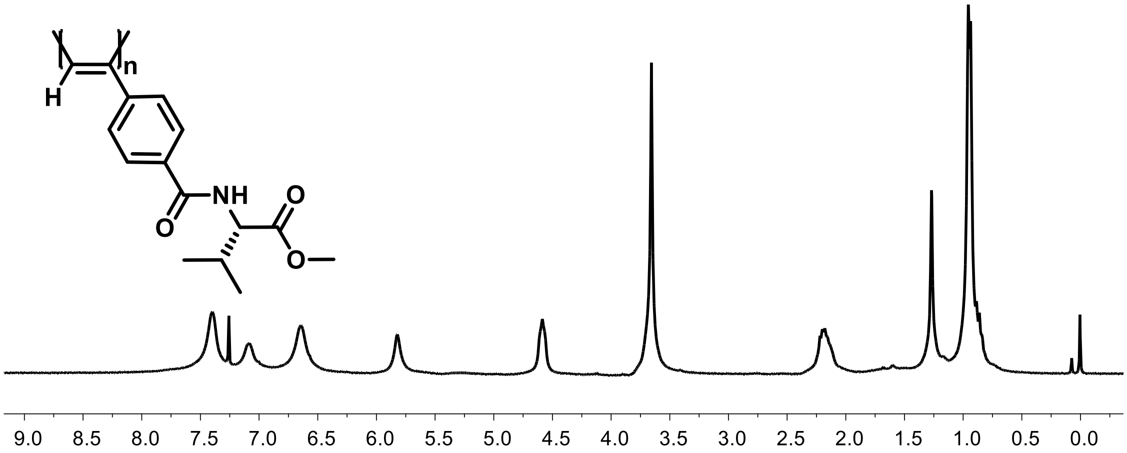

Supplementary Figure 7. ^{1}H NMR spectra of poly-(*S*)-**6** ($CDCl_3$, 300 MHz).

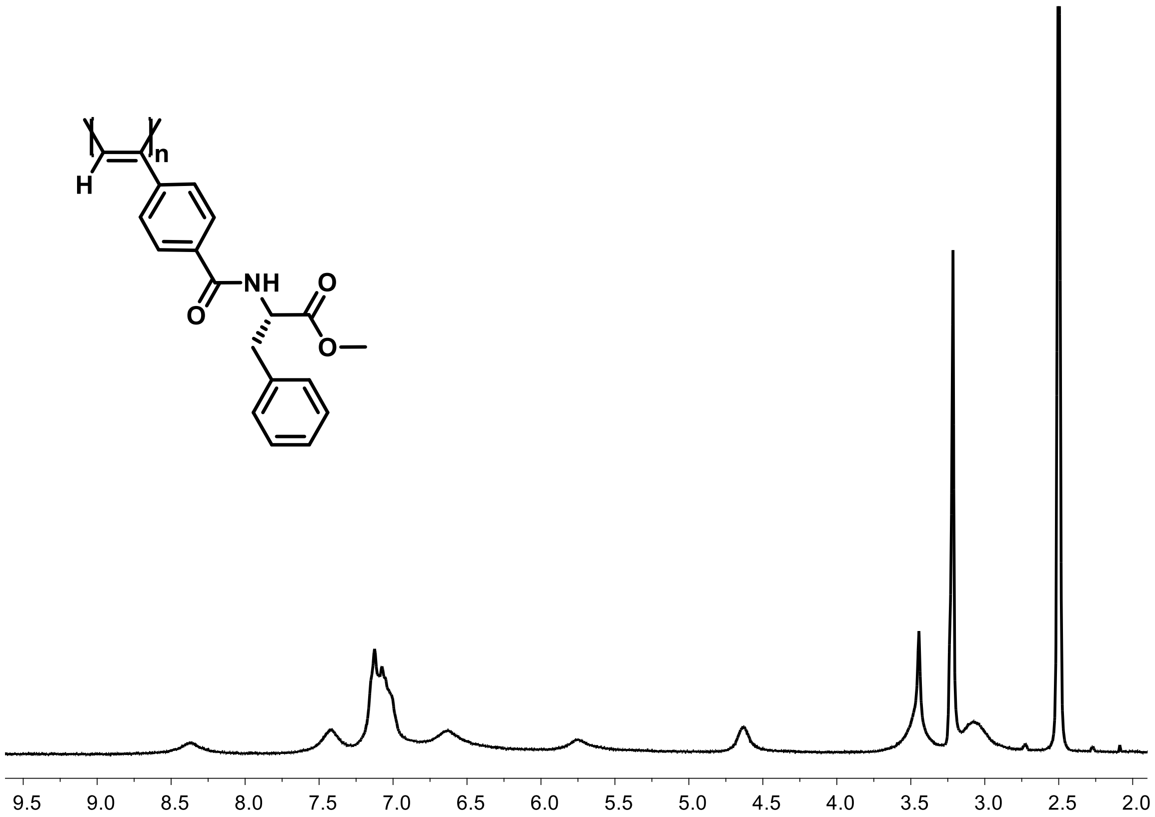

Supplementary Figure 8. ^{1}H NMR spectra of poly-(*S*)-**7** (DMSO, 300 MHz).

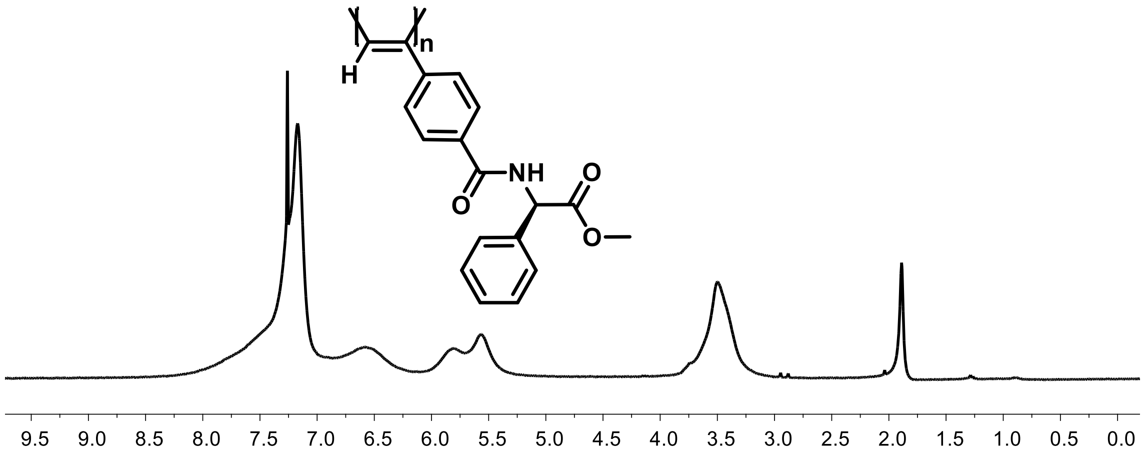

Supplementary Figure 9. ^{1}H NMR spectra of poly-(*R*)-**8** ($CDCl_3$, 300 MHz).

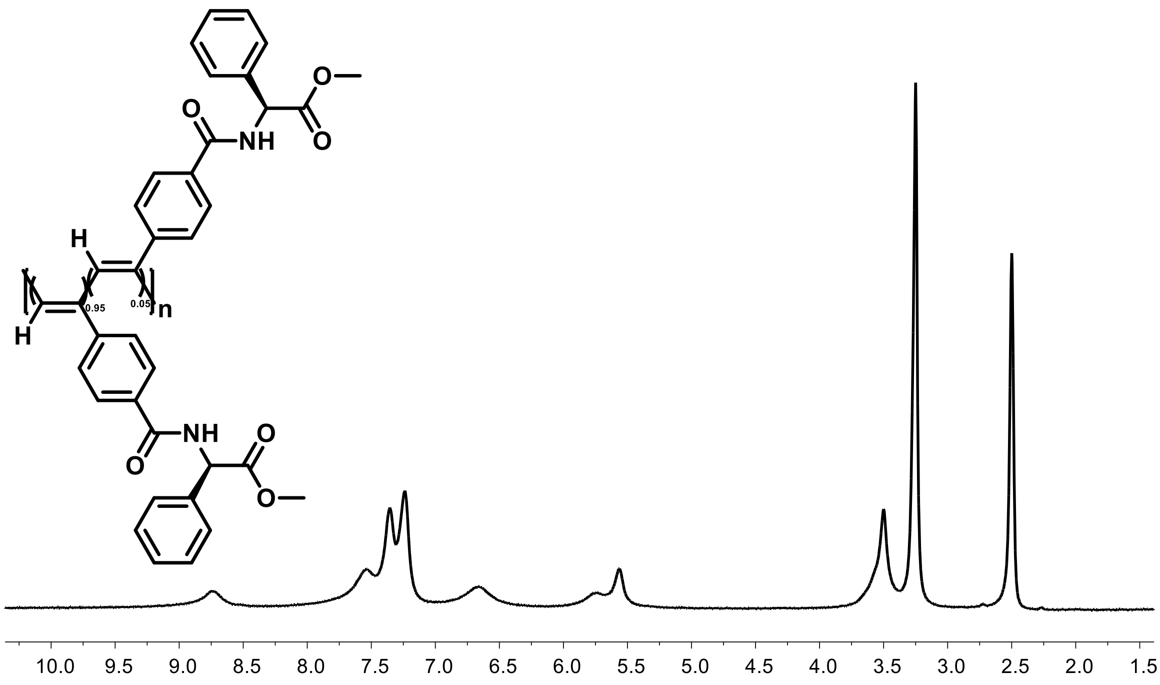

Supplementary Figure 10. ^{1}H NMR spectra of poly[(*R*)-**1**$_{0.95}$-co-(*S*)-**1**$_{0.05}$] (DMSO, 300 MHz).

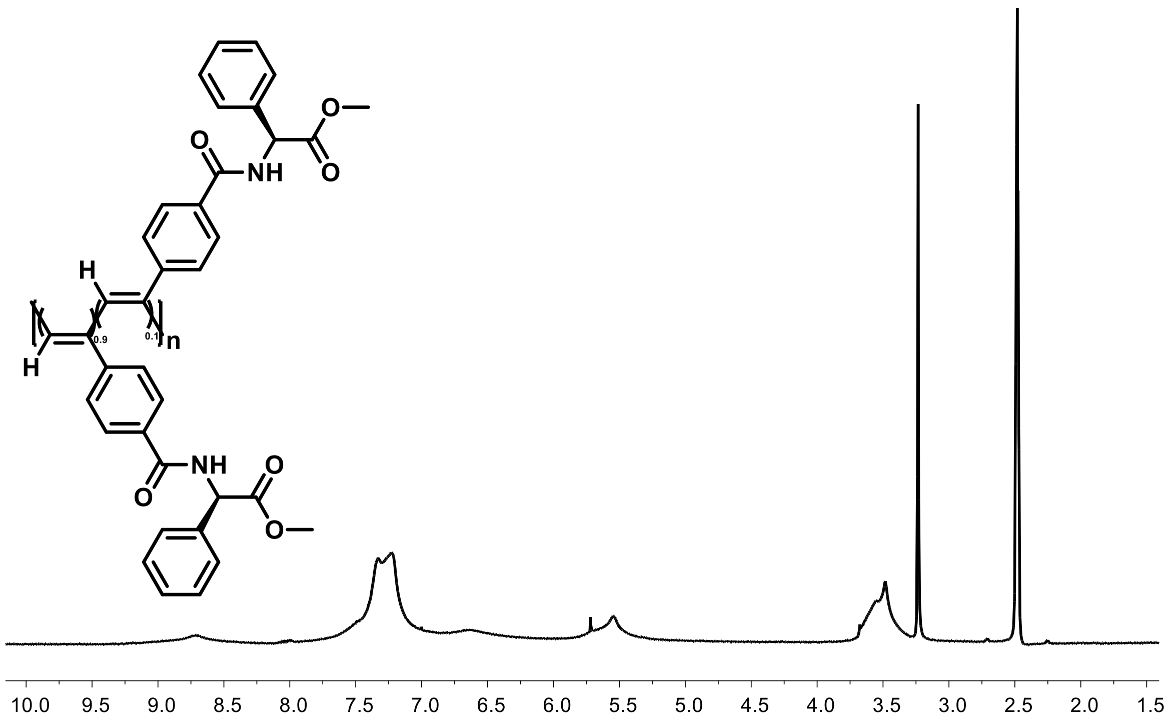

Supplementary Figure 11. ^{1}H NMR spectra of poly[(*R*)-**1**$_{0.9}$-co-(*S*)-**1**$_{0.1}$] (DMSO, 300 MHz).

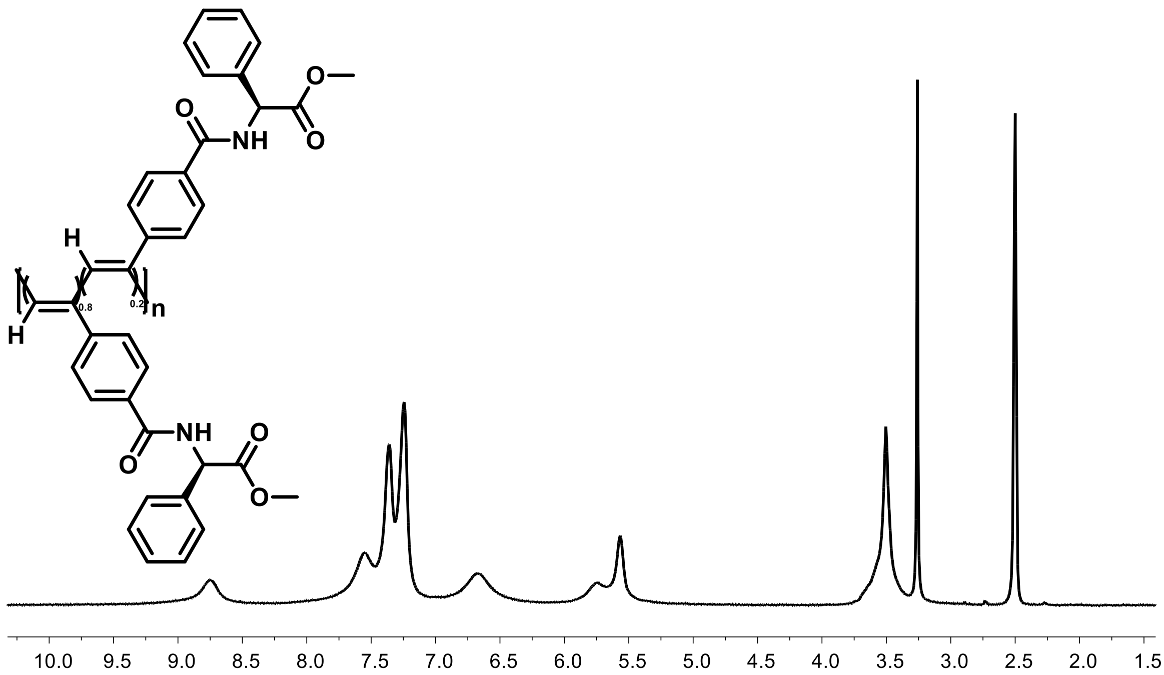

Supplementary Figure 12. ^{1}H NMR spectra of poly[(*R*)-**1**$_{0.8}$-co-(*S*)-**1**$_{0.2}$] (DMSO, 300 MHz).

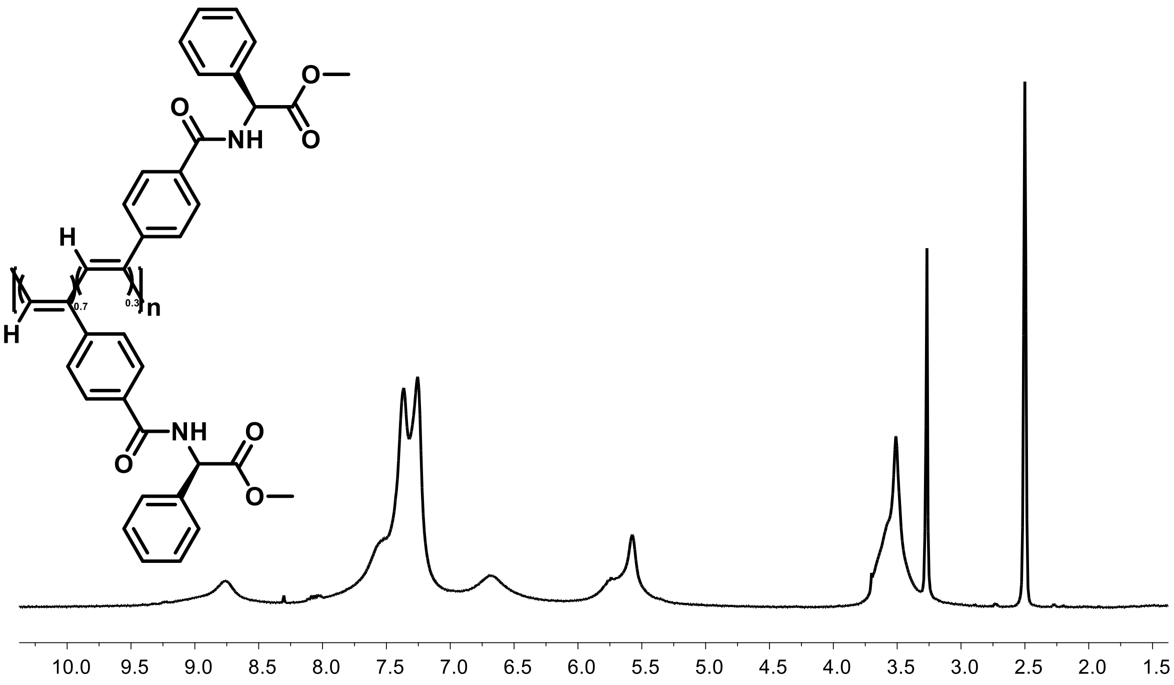

Supplementary Figure 13. ^{1}H NMR spectra of poly[(*R*)-**1**$_{0.7}$-co-(*S*)-**1**$_{0.3}$] (DMSO, 300 MHz).

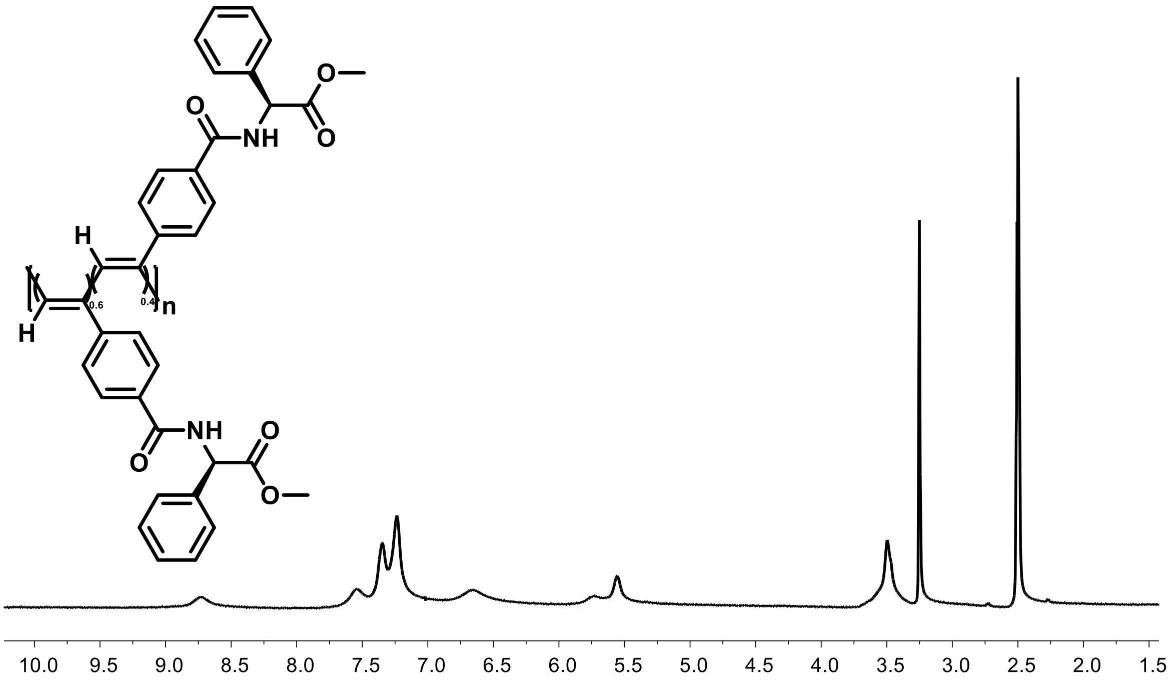

Supplementary Figure 14. ^{1}H NMR spectra of poly[(*R*)-**1**$_{0.6}$-co-(*S*)-**1**$_{0.4}$] (DMSO, 300 MHz).

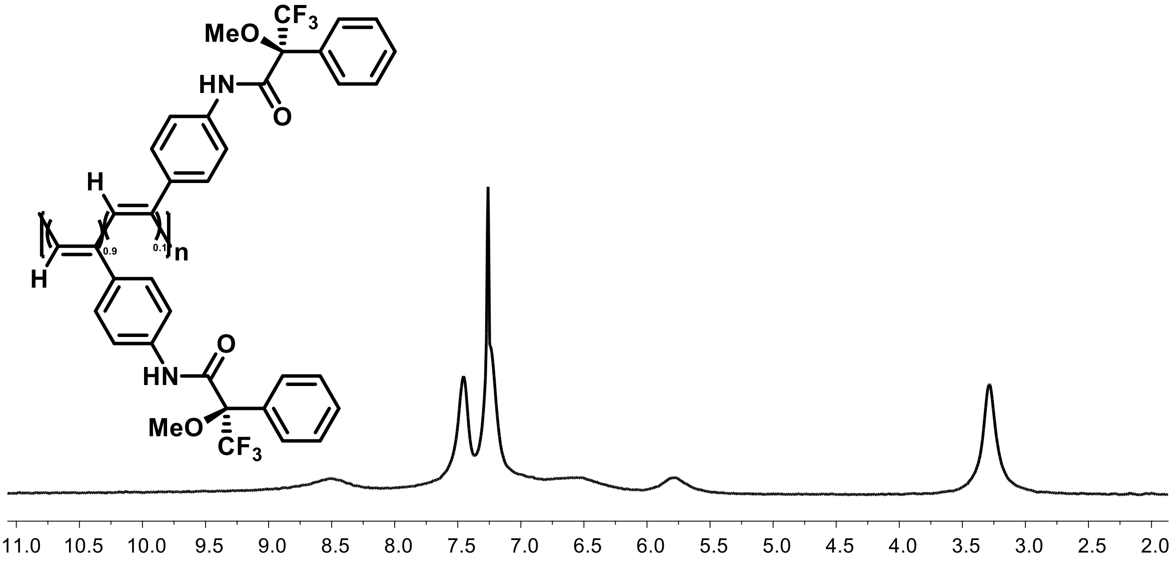

Supplementary Figure 15. ^{1}H NMR spectra of poly[(*R*)-**2**$_{0.9}$-co-(*S*)-**2**$_{0.1}$] ($CDCl_3$, 300 MHz).

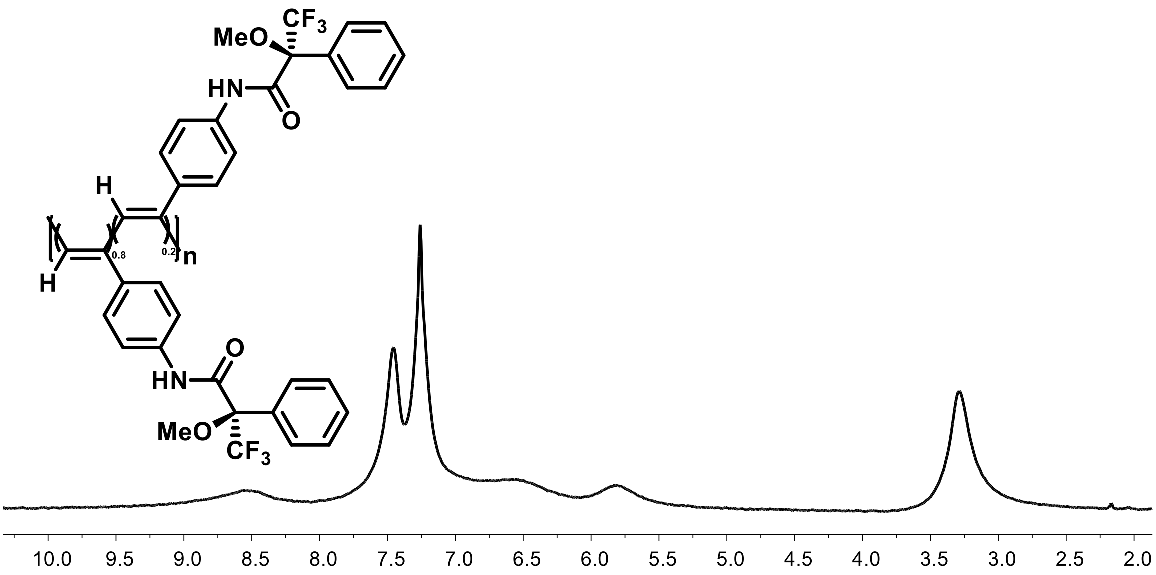

Supplementary Figure 16. ^{1}H NMR spectra of poly[(*R*)-**2**$_{0.8}$-co-(*S*)-**2**$_{0.2}$] ($CDCl_3$, 300 MHz).

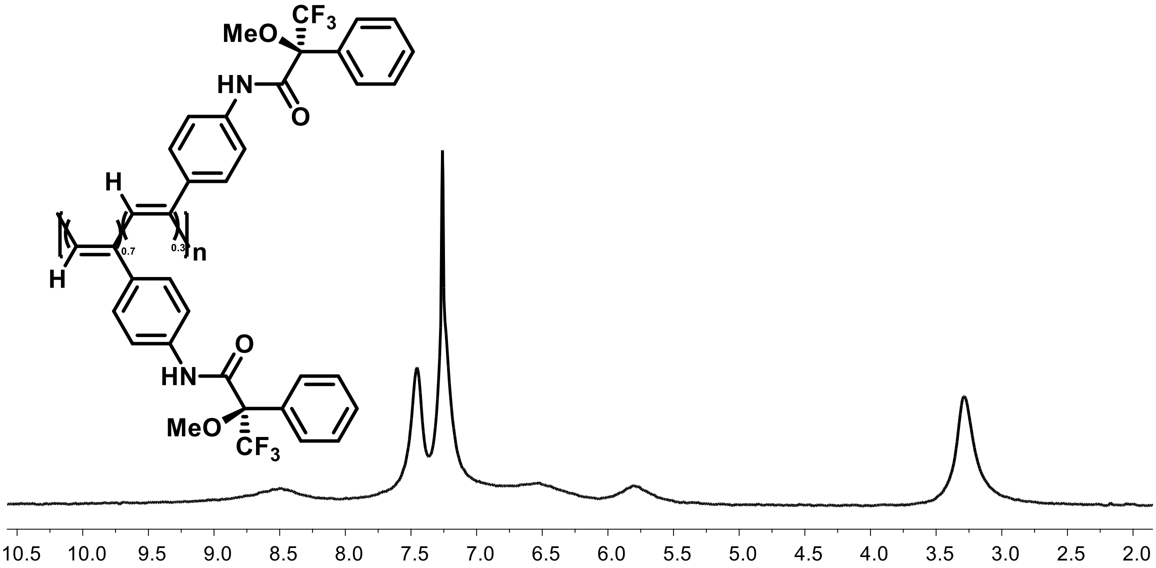

Supplementary Figure 17. ^{1}H NMR spectra of poly[(*R*)-**2**$_{0.7}$-co-(*S*)-**2**$_{0.3}$] ($CDCl_3$, 300 MHz).

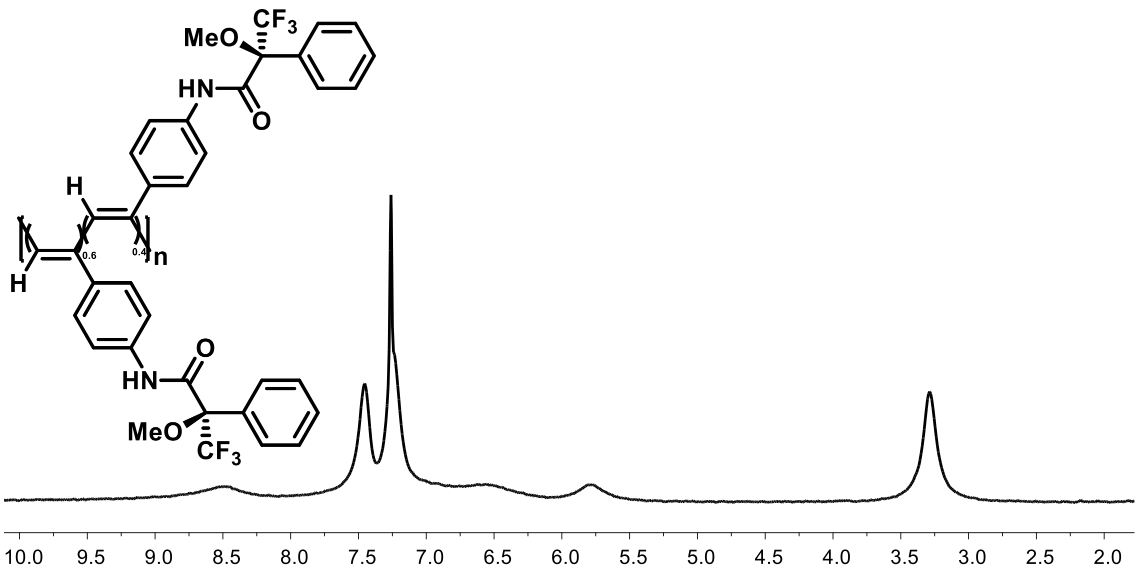

Supplementary Figure 18. ^{1}H NMR spectra of poly[(*R*)-$\mathbf{2}_{0.6}$-co-(*S*)-$\mathbf{2}_{0.4}$] ($CDCl_3$, 300 MHz).

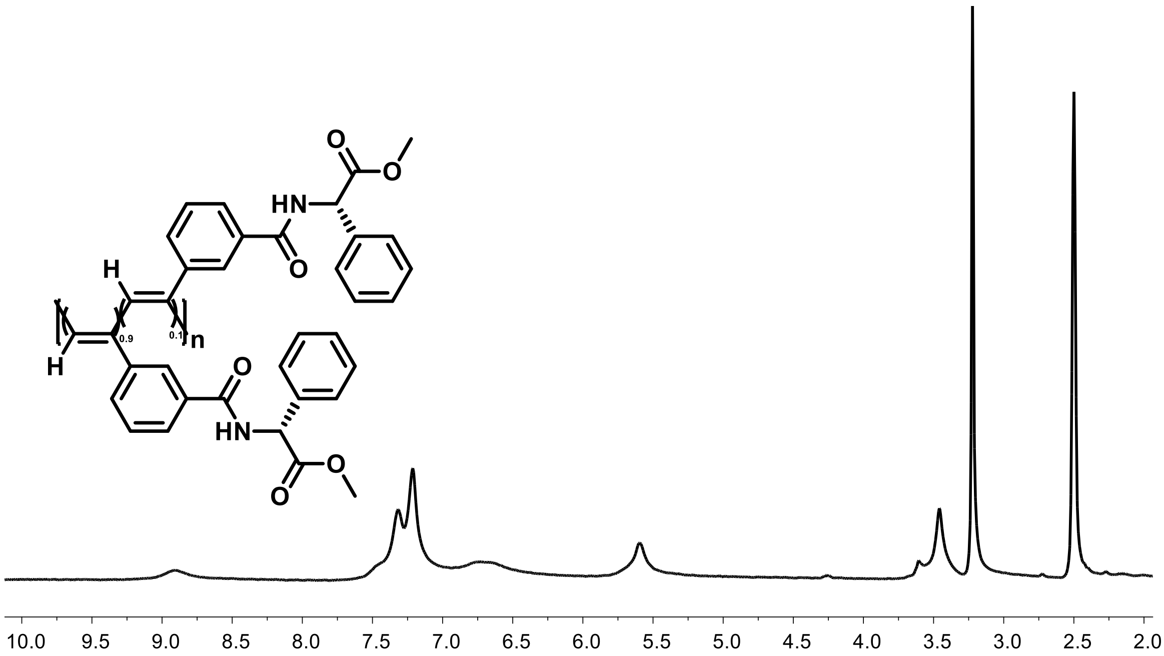

Supplementary Figure 19. ^{1}H NMR spectra of poly[(*R*)-**8**$_{0.9}$-co-(*S*)-**8**$_{0.1}$] (DMSO, 300 MHz).

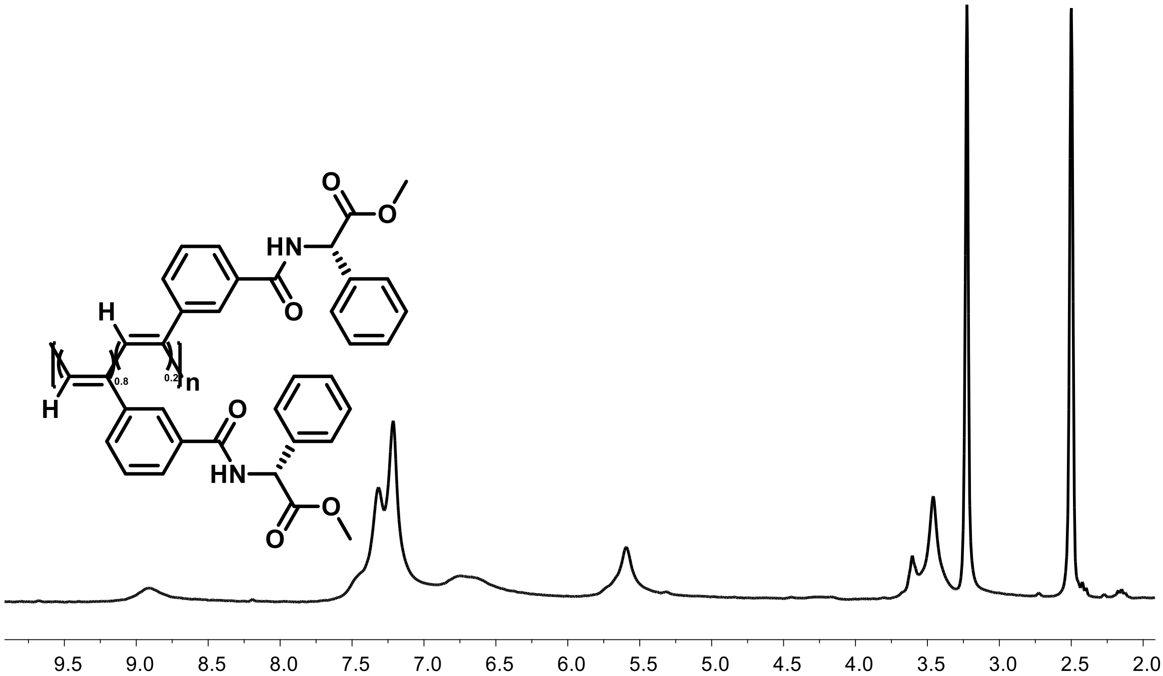

Supplementary Figure 20. ^{1}H NMR spectra of poly[(*R*)-**8**$_{0.8}$-co-(*S*)-**8**$_{0.2}$] (DMSO, 300 MHz).

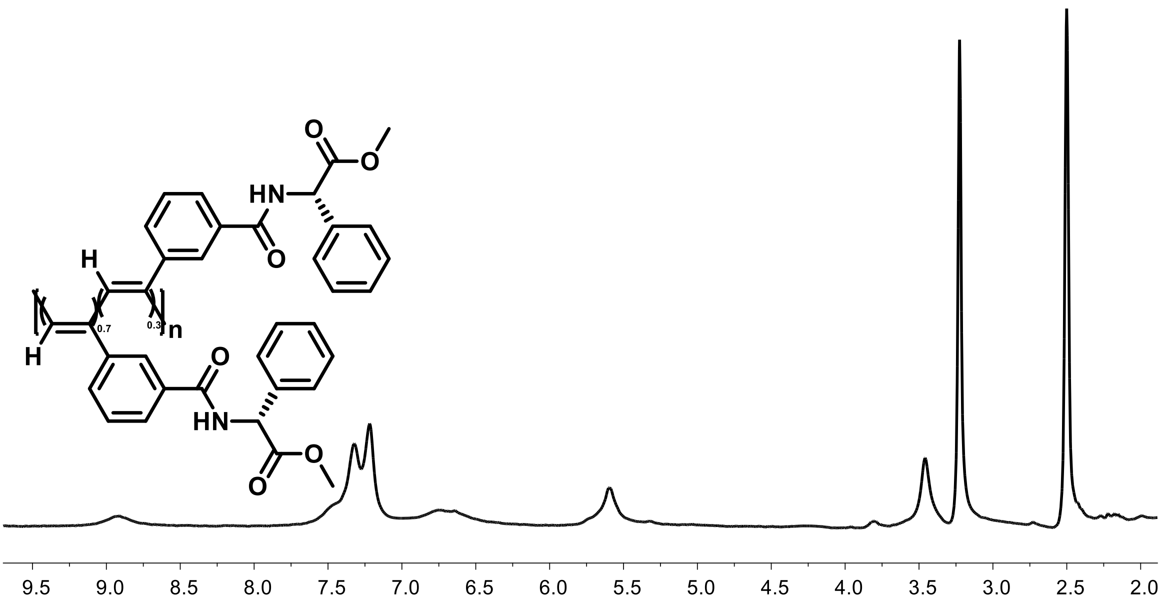

Supplementary Figure 21. ^{1}H NMR spectra of poly[(*R*)-**8**$_{0.7}$-co-(*S*)-**8**$_{0.3}$] (DMSO, 300 MHz).

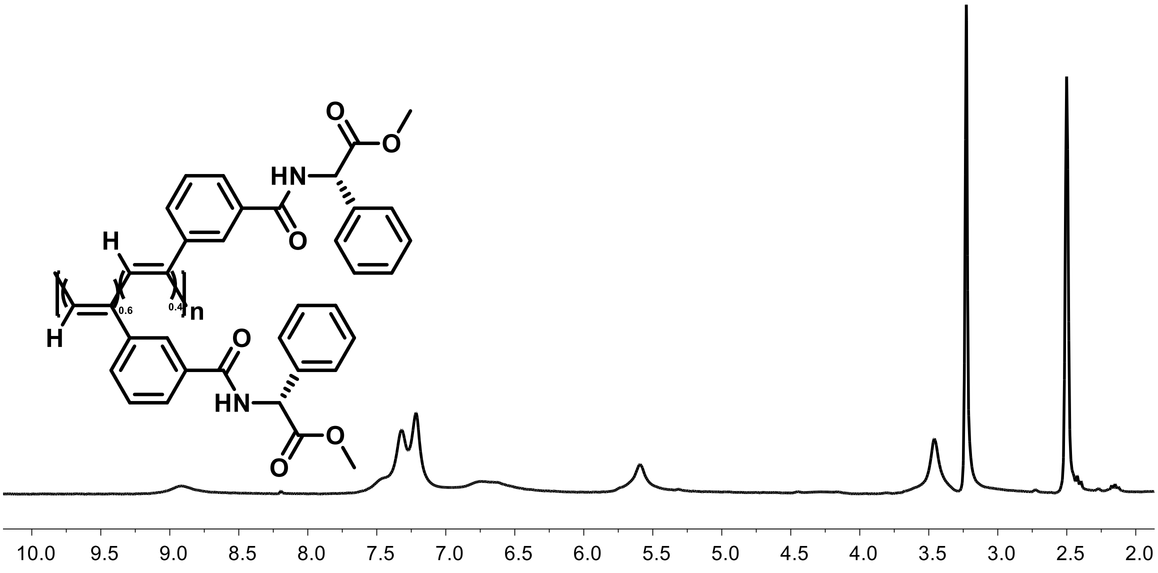

Supplementary Figure 22. ^{1}H NMR spectra of poly[(*R*)-**8**$_{0.6}$-co-(*S*)-**8**$_{0.4}$] (DMSO, 300 MHz).

Poly-(R)-**1**

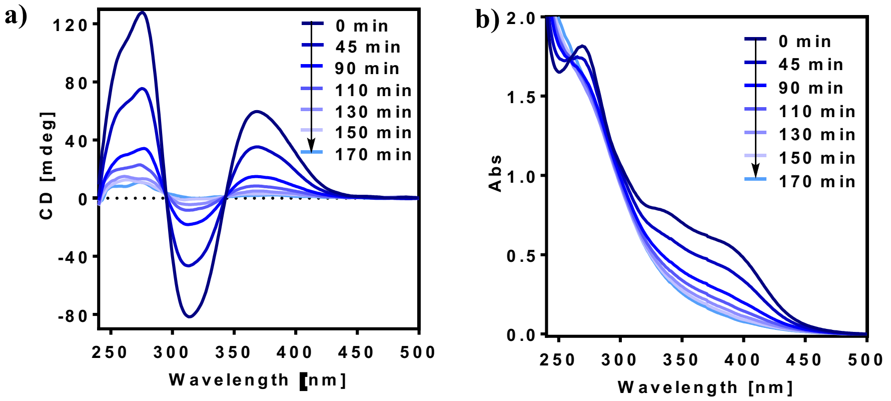

Supplementary Figure 23. (a) CD and (b) UV spectra of poly-(R)-**1** in $CHCl_3$ after irradiation under visible light. [poly-(R)-**1**]= 1.02·10^{-3} M.

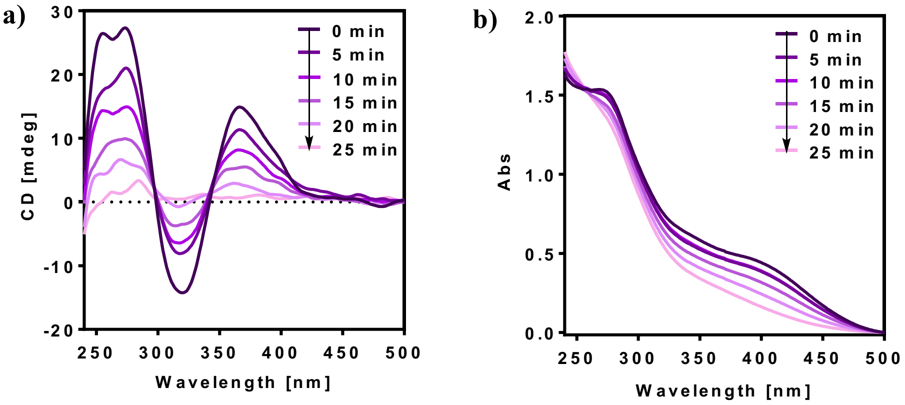

Supplementary Figure 24. (a) CD and (b) UV spectra of poly-(R)-**1** in THF after irradiation under visible light. [poly-(R)-**1**]= 1.02·10^{-3} M.

Poly-(R)-**2**

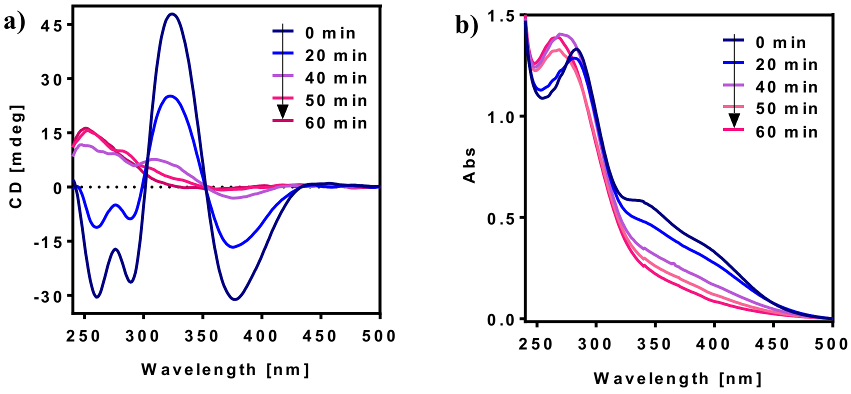

Supplementary Figure 25. (a) CD and (b) UV spectra poly-(R)-**2** in $CHCl_3$ after irradiation under visible light. [poly-(R)-**2**]= 9.00·10^{-4} M.

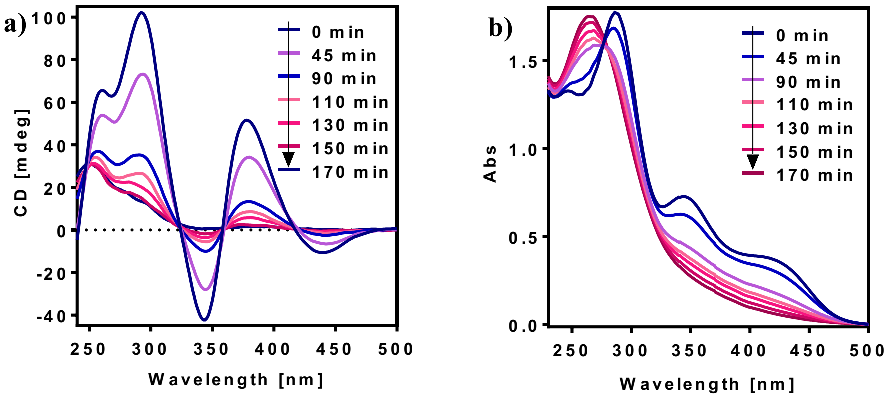

Supplementary Figure 26. (a) CD and (b) UV spectra poly-(*R*)-**2** in THF after irradiation under visible light. [poly-(*R*)-**2**]= $9.00 \cdot 10^{-4}$ M.

Poly-(*S*)-**3**

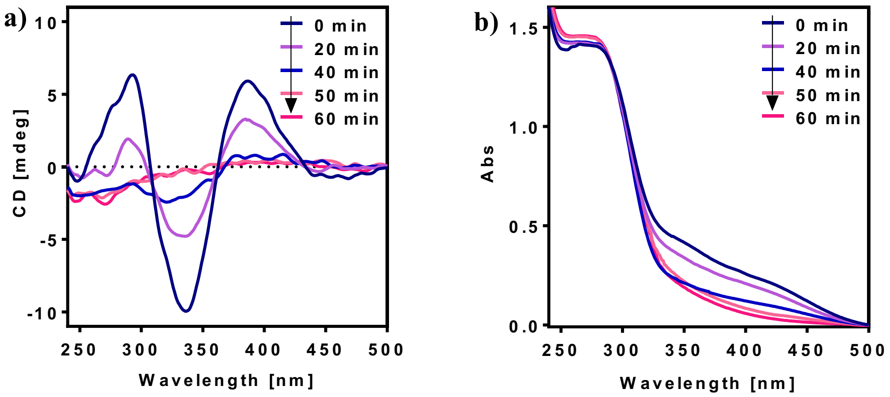

Supplementary Figure 27. (a) CD and (b) UV spectra of poly-(*S*)-**3** in THF after irradiation under visible light. [poly-(*S*)-**3**]= $1.19 \cdot 10^{-3}$ M.

Poly-(*R*)-**4**

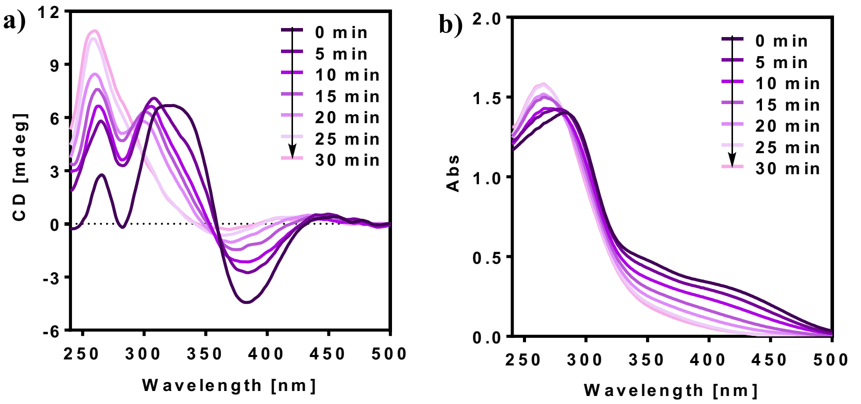

Supplementary Figure 28. (a) CD and (b) UV spectra of poly-(*R*)-**4** in $CHCl_3$ after irradiation under visible light. [poly-(*R*)-**4**]= $1.13 \cdot 10^{-3}$ M.

Poly-(S)-**5**

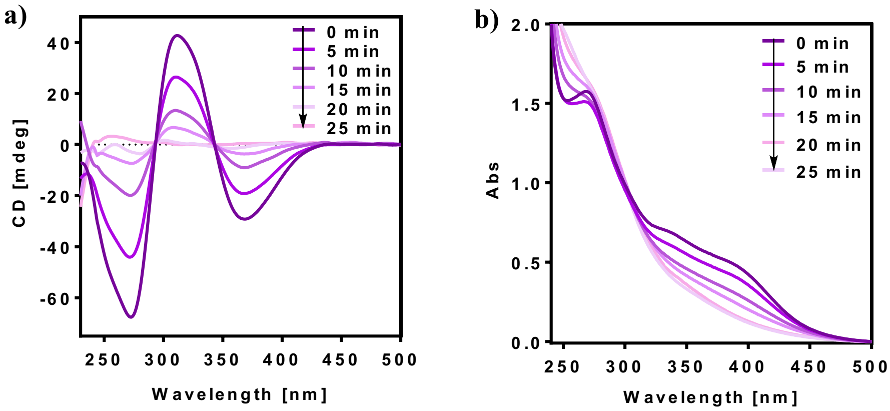

Supplementary Figure 29. (a) CD and (b) UV spectra of poly-(S)-**5** in $CHCl_3$ after irradiation under visible light. [poly-(S)-**5**]= $1.30 \cdot 10^{-3}$ M.

Poly-(S)-**6**

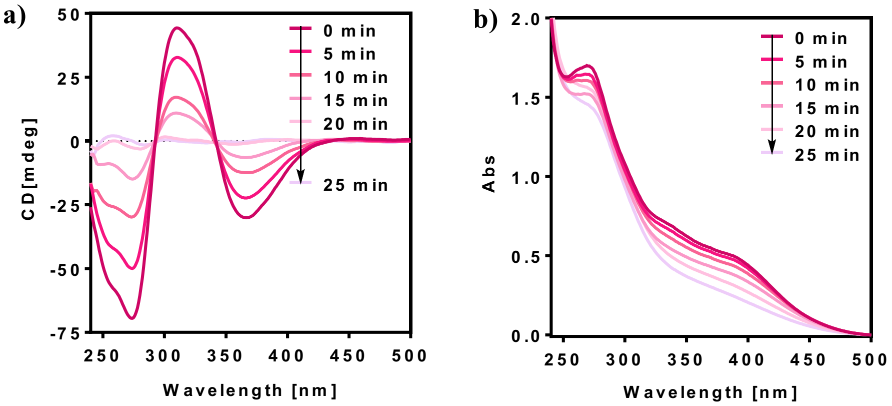

Supplementary Figure 30. (a) CD and (b) UV spectra of poly-(S)-**6** in $CHCl_3$ after irradiation under visible light. [poly-(S)-**6**]= $1.16 \cdot 10^{-3}$ M.

Poly-(S)-**7**

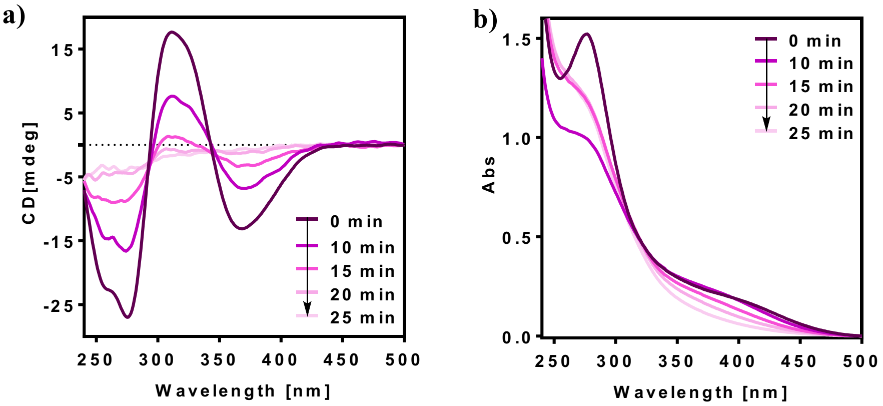

Supplementary Figure 31. (a) CD and (b) UV spectra of poly-(S)-**7** in $CHCl_3$ after irradiation under visible light. [poly-(S)-**7**]= $9.76 \cdot 10^{-4}$ M.

Poly-(*R*)-**8**

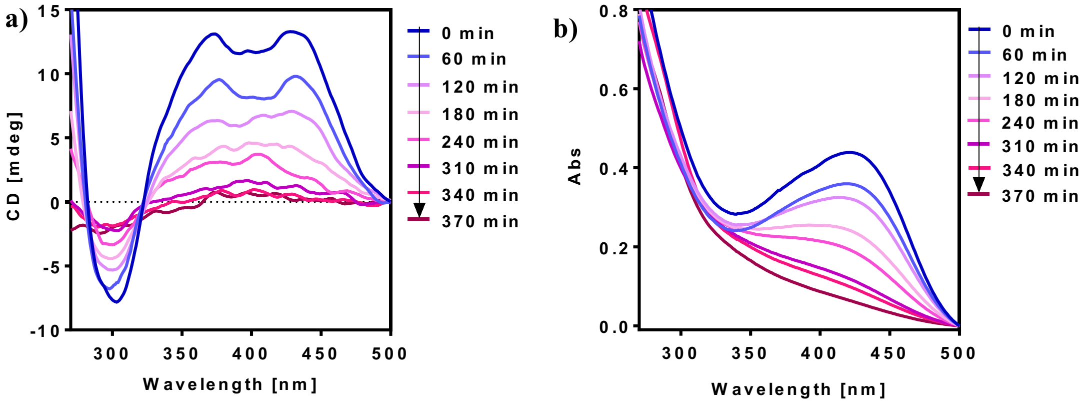

Supplementary Figure 32. (a) CD and (b) UV spectra of poly-(*R*)-**8** in $CHCl_3$ after irradiation under visible light. [poly-(*R*)-**8**]= $1.02 \cdot 10^{-3}$ M.

Poly-(*R*)-**1**

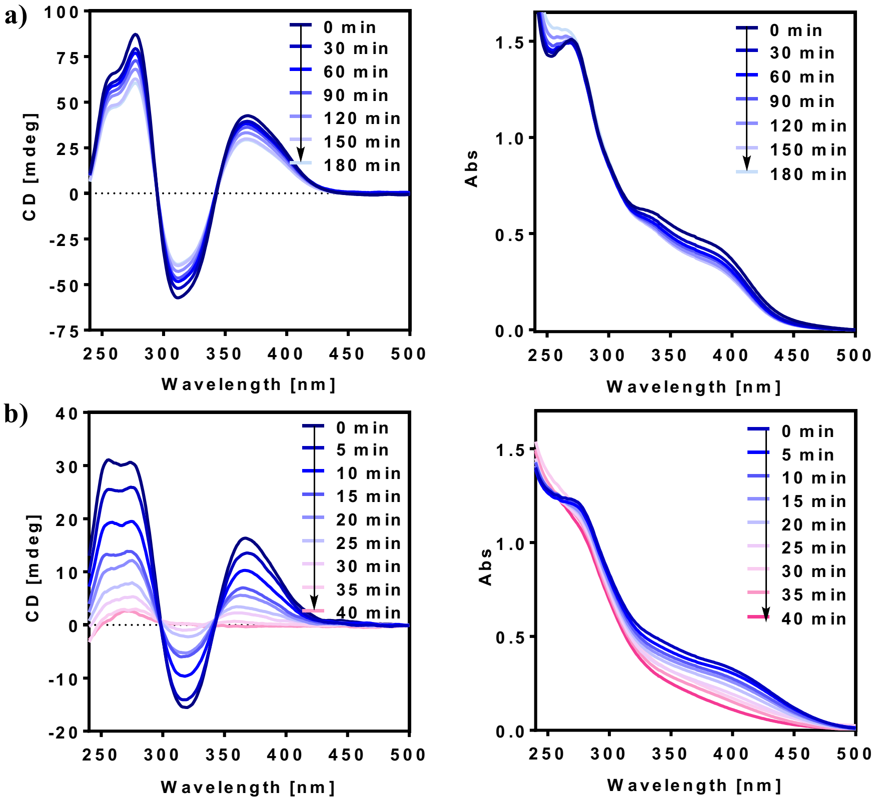

Supplementary Figure 33. (a) CD and UV spectra of poly-(*R*)-**1** after irradiation under visible light at 4 °C in a) $CHCl_3$ and b) THF. [poly-(*R*)-**1**]= $1.02 \cdot 10^{-3}$ M.

Poly-(*R*)-**2**

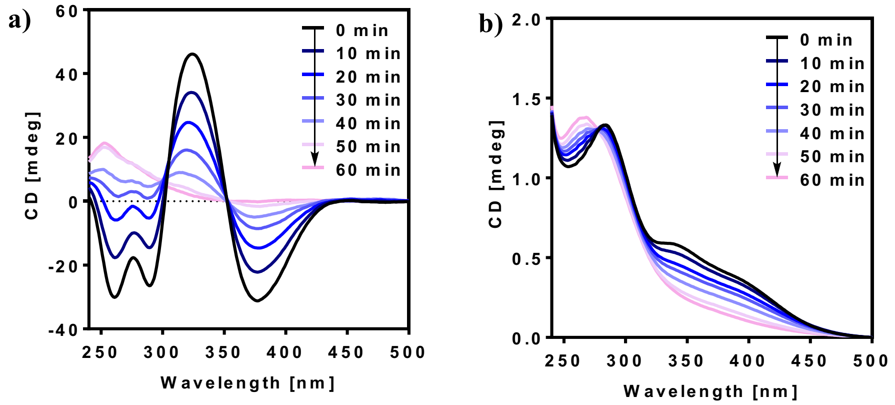

Supplementary Figure 34. (a) CD and (b) UV spectra poly-(*R*)-**2** in $CHCl_3$ after irradiation under visible light at 4 °C. [poly-(*R*)-**2**]= $9.00 \cdot 10^{-4}$ M.

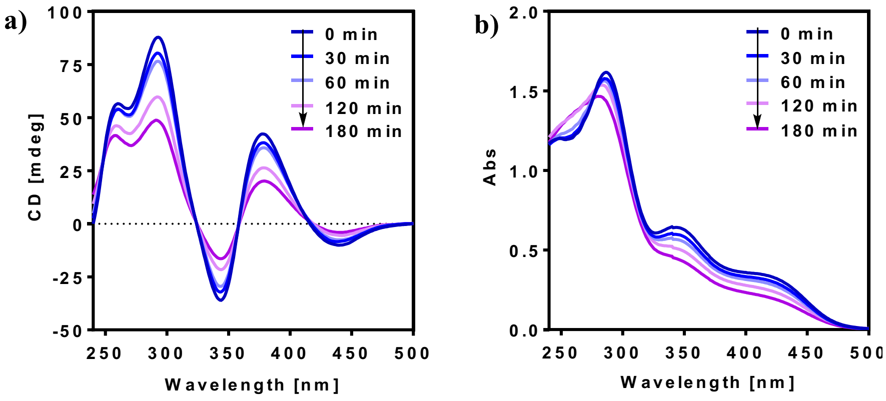

Supplementary Figure 35. (a) CD and (b) UV spectra poly-(*R*)-**2** in THF after irradiation under visible light at 4 °C. [poly-(*R*)-**2**]= 9.00·10^{-4} M.

Poly-(*S*)-**3**

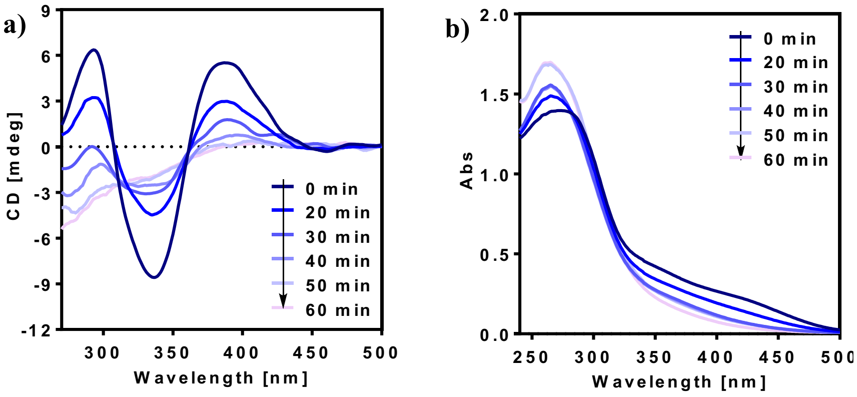

Supplementary Figure 36. (a) CD and (b) UV spectra of poly-(*S*)-**3** in THF after irradiation under visible light at 4 °C. [poly-(*S*)-**3**]= 1.19·10^{-3} M.

Poly-(*R*)-**4**

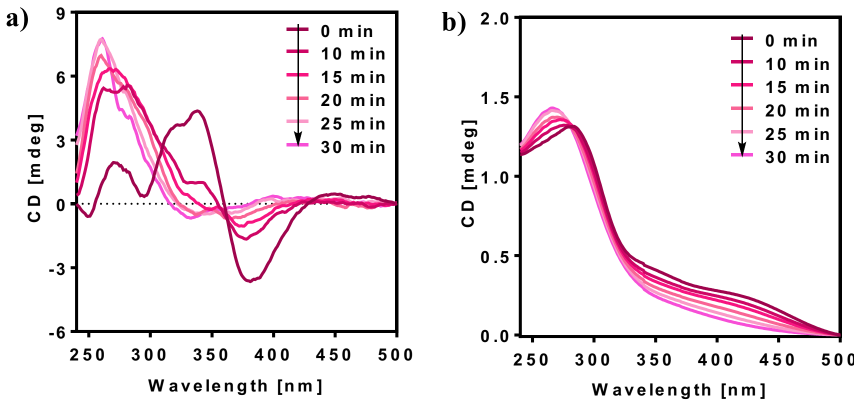

Supplementary Figure 37. (a) CD and (b) UV spectra of poly-(*S*)-**5** in THF after irradiation under visible light at 4 °C. [poly-(*R*)-**4**]= 1.13·10^{-3} M.

Poly-(*S*)-**5**

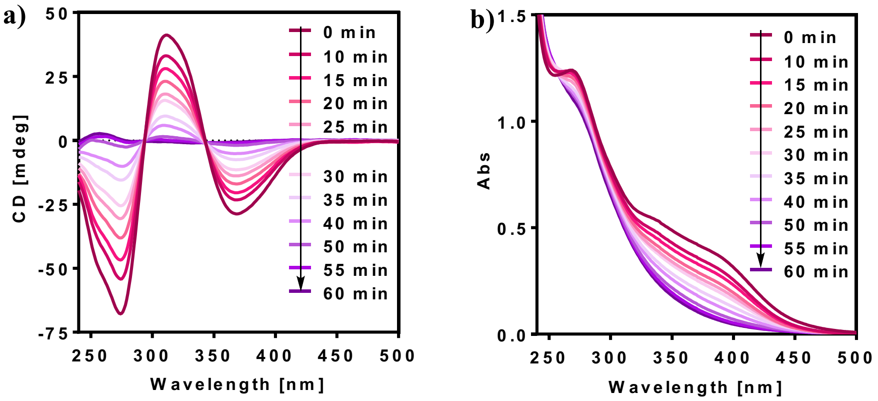

Supplementary Figure 38. (a) CD and (b) UV spectra of poly-(*S*)-**6** in $CHCl_3$ after irradiation under visible light at 4 °C. [poly-(*S*)-**5**]= $1.30\cdot10^{-3}$ M.

Poly-(*S*)-**6**

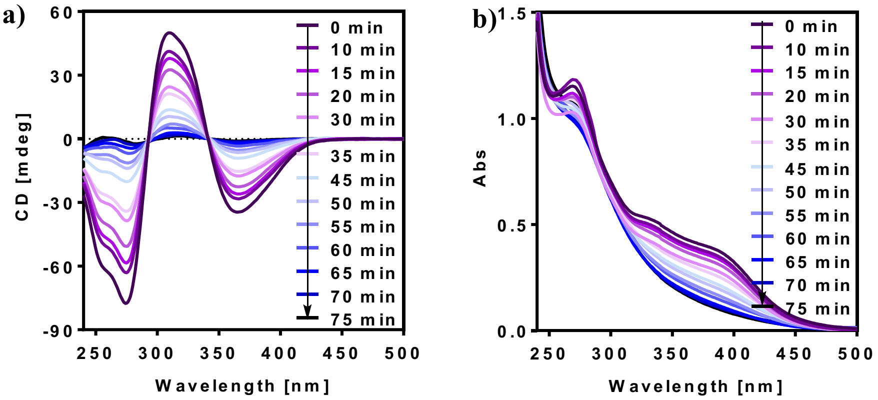

Supplementary Figure 39. (a) CD and (b) UV spectra of poly-(*S*)-**7** in $CHCl_3$ after irradiation under visible light at 4 °C. [poly-(*S*)-**6**]= $1.16\cdot10^{-3}$ M.

Poly-(S)-**7**

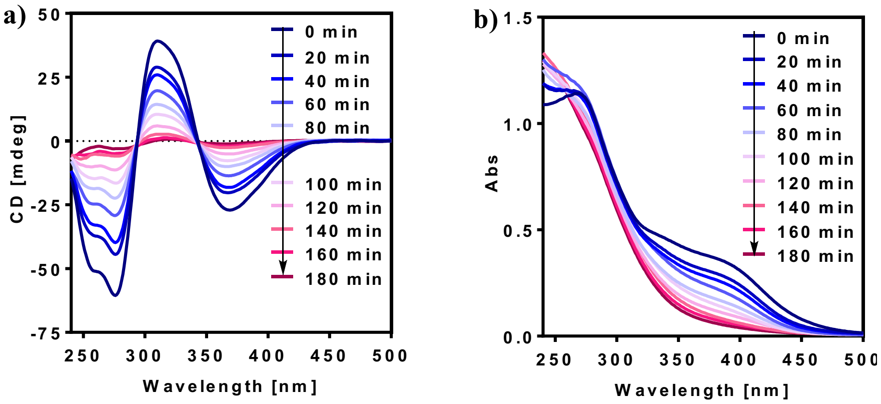

Supplementary Figure 40. (a) CD and (b) UV spectra of poly-(S)-**8** in $CHCl_3$ after irradiation under visible light at 4 °C. [poly-(S)-**7**]= $9.76{\cdot}10^{-4}$ M.

Poly[(*R*)-**1**$_{r}$-co-(*S*)-**1**$_{(1-r)}$] series

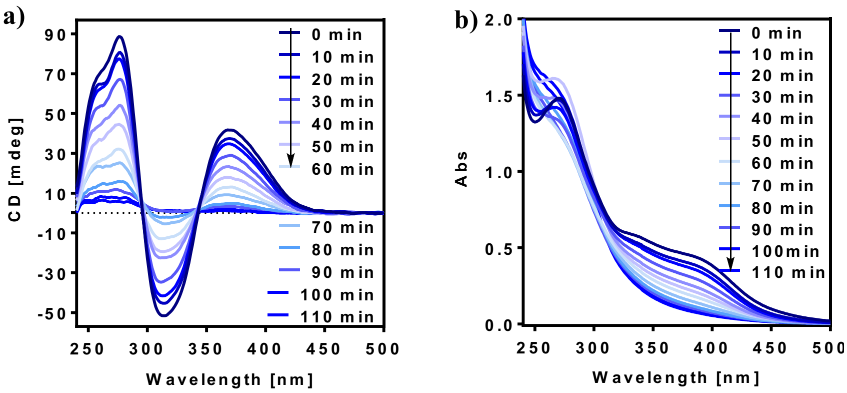

Supplementary Figure 41. (a) CD and (b) UV spectra of poly[(*R*)-**1**$_{0.95}$-co-(*S*)-**1**$_{0.05}$] in $CHCl_3$ after irradiation under visible light. [poly[(*R*)-**1**$_{0.95}$-co-(*S*)-**1**$_{0.05}$]]= $1.02 \cdot 10^{-3}$ M.

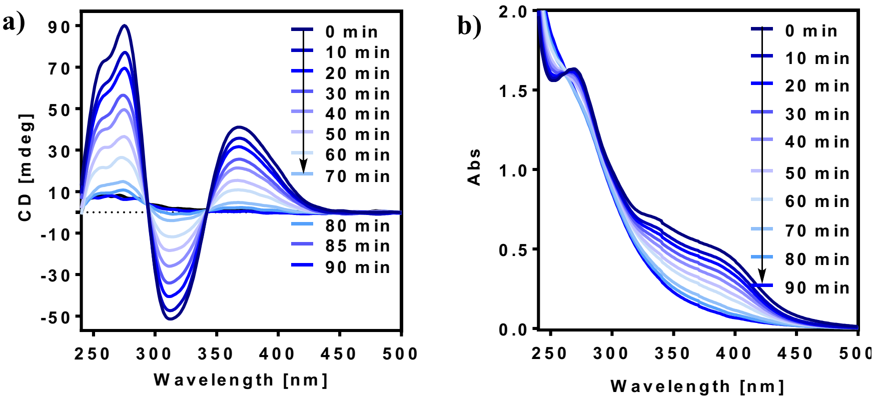

Supplementary Figure 42. (a) CD and (b) UV spectra of poly[(*R*)-**1**$_{0.9}$-co-(*S*)-**1**$_{0.1}$] in $CHCl_3$ after irradiation under visible light. [poly[(*R*)-**1**$_{0.9}$-co-(*S*)-**1**$_{0.1}$]]= $1.02 \cdot 10^{-3}$ M.

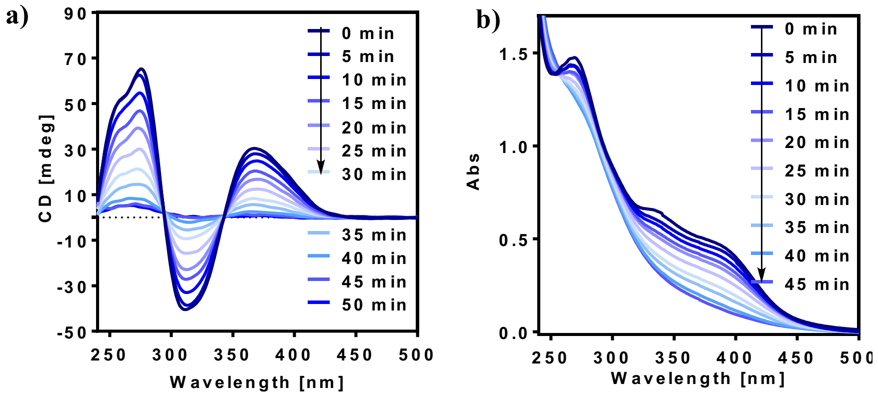

Supplementary Figure 43. (a) CD and (b) UV spectra of poly[(*R*)-**1**$_{0.8}$-co-(*S*)-**1**$_{0.2}$] in $CHCl_3$ after irradiation under visible light. [poly[(*R*)-**1**$_{0.8}$-co-(*S*)-**1**$_{0.2}$]]= $1.02\cdot10^{-3}$ M.

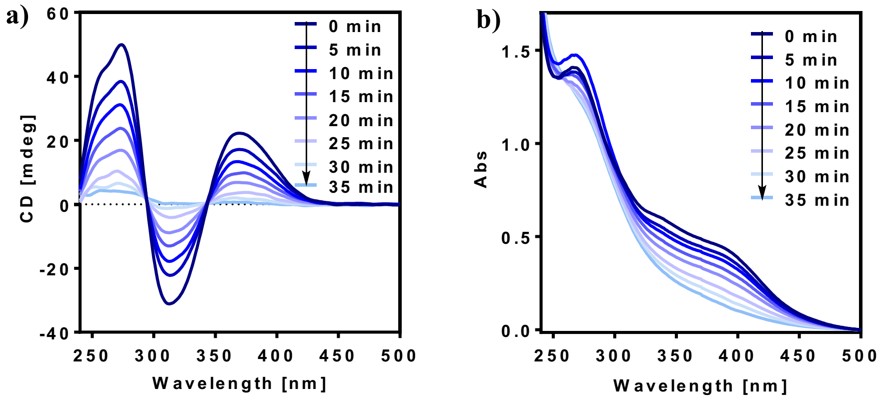

Supplementary Figure 44. (a) CD and (b) UV spectra of poly[(*R*)-**1**$_{0.7}$-co-(*S*)-**1**$_{0.3}$] in $CHCl_3$ after irradiation under visible light. [poly[(*R*)-**1**$_{0.7}$-co-(*S*)-**1**$_{0.3}$]]= $1.02\cdot10^{-3}$ M.

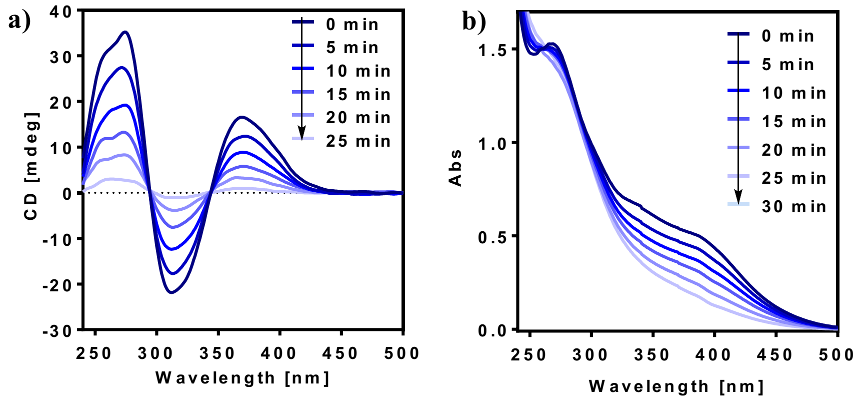

Supplementary Figure 45. (a) CD and (b) UV spectra of poly[(*R*)-**1**$_{0.6}$-co-(*S*)-**1**$_{0.4}$] in $CHCl_3$ after irradiation under visible light. [poly[(*R*)-**1**$_{0.6}$-co-(*S*)-**1**$_{0.4}$]]= $1.02\cdot10^{-3}$ M.

Poly[(*R*)-**2**$_r$-co-(*S*)-**2**$_{(1-r)}$] series

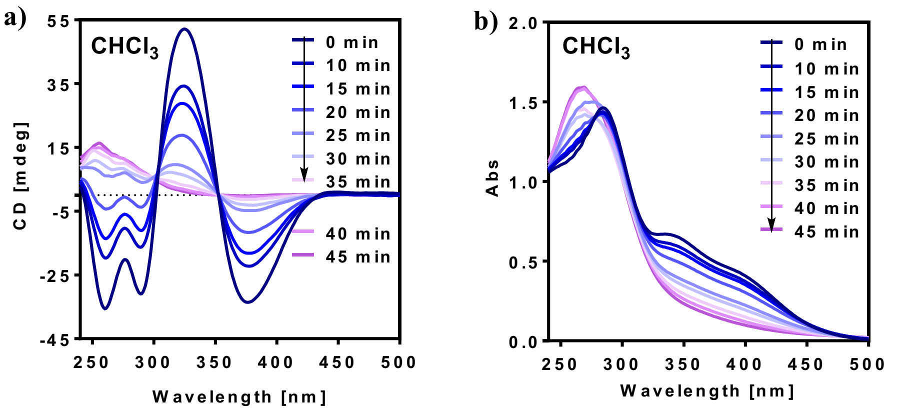

Supplementary Figure 46. (a) CD and (b) UV spectra of poly[(*R*)-**2**$_{0.9}$-co-(*S*)-**2**$_{0.1}$] in $CHCl_3$ after irradiation under visible light. [poly[(*R*)-**2**$_{0.9}$-co-(*S*)-**2**$_{0.1}$]]= $9.00 \cdot 10^{-4}$ M.

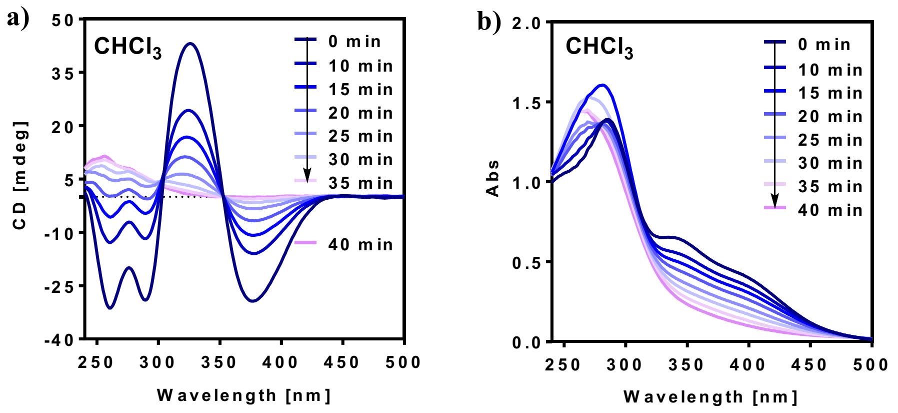

Supplementary Figure 47. (a) CD and (b) UV spectra of poly[(*R*)-**2**$_{0.8}$-co-(*S*)-**2**$_{0.2}$] in $CHCl_3$ after irradiation under visible light. [poly[(*R*)-**2**$_{0.8}$-co-(*S*)-**2**$_{0.2}$]]= $9.00 \cdot 10^{-4}$ M.

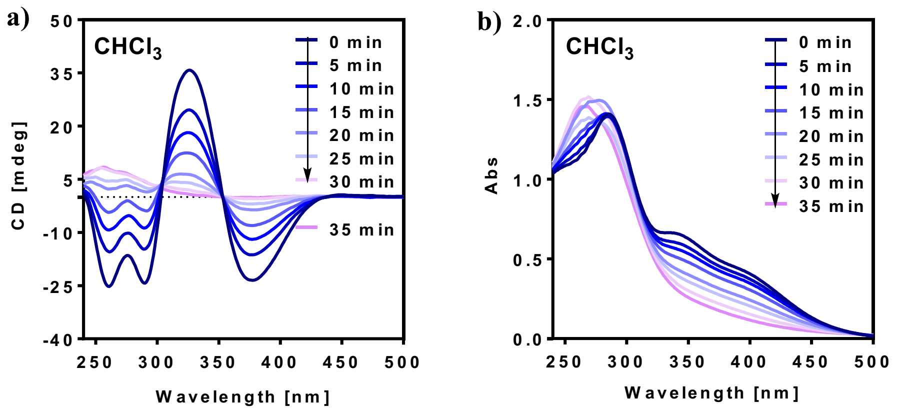

Supplementary Figure 48. (a) CD and (b) UV spectra of poly[(*R*)-**2**$_{0.7}$-co-(*S*)-**2**$_{0.3}$] in $CHCl_3$ after irradiation under visible light. [poly[(*R*)-**2**$_{0.7}$-co-(*S*)-**2**$_{0.3}$]]= $9.00 \cdot 10^{-4}$ M.

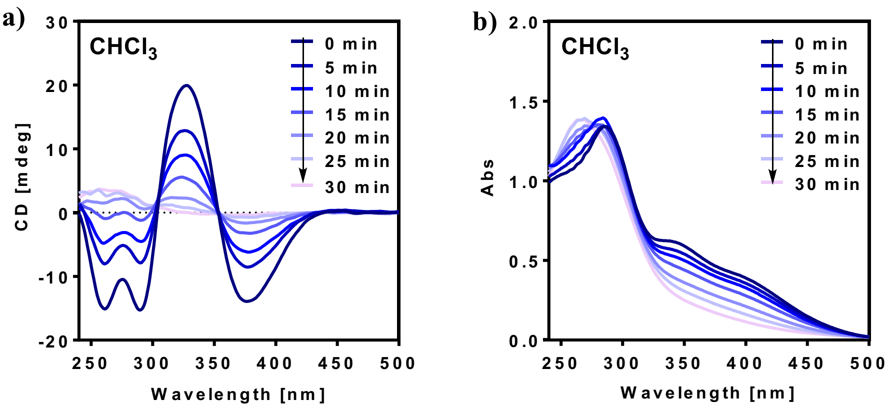

Supplementary Figure 49. (a) CD and (b) UV spectra of poly[(*R*)-**2**$_{0.6}$-co-(*S*)-**2**$_{0.4}$] in $CHCl_3$ after irradiation under visible light. [poly[(*R*)-**2**$_{0.6}$-co-(*S*)-**2**$_{0.4}$]]= 9.00·10^{-4} M.

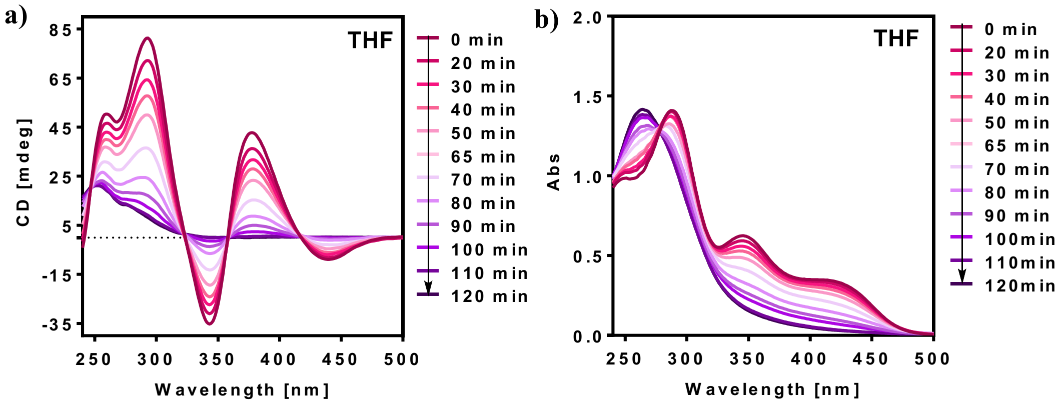

Supplementary Figure 50. (a) CD and (b) UV spectra of poly[(*R*)-**2**$_{0.9}$-co-(*S*)-**2**$_{0.1}$] in THF after irradiation under visible light. [poly[(*R*)-**2**$_{0.9}$-co-(*S*)-**2**$_{0.1}$]]= 9.00·10^{-4} M.

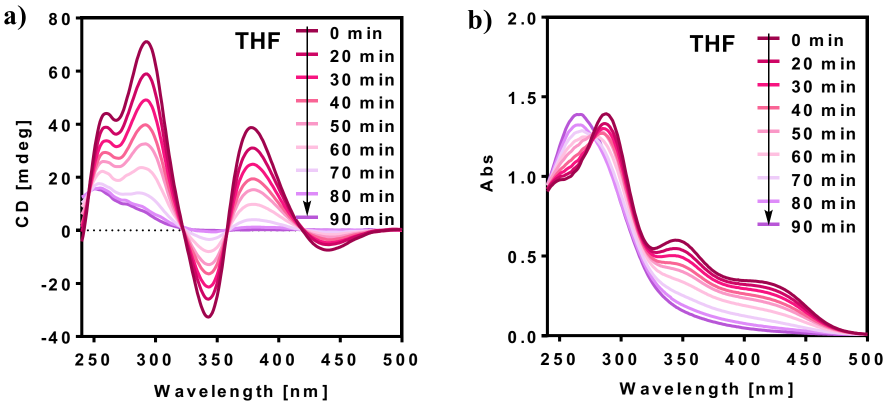

Supplementary Figure 51. (a) CD and (b) UV spectra of poly[(*R*)-**2**$_{0.8}$-co-(*S*)-**2**$_{0.2}$] in THF after irradiation under visible light. [poly[(*R*)-**2**$_{0.8}$-co-(*S*)-**2**$_{0.2}$]]= 9.00·10^{-4} M.

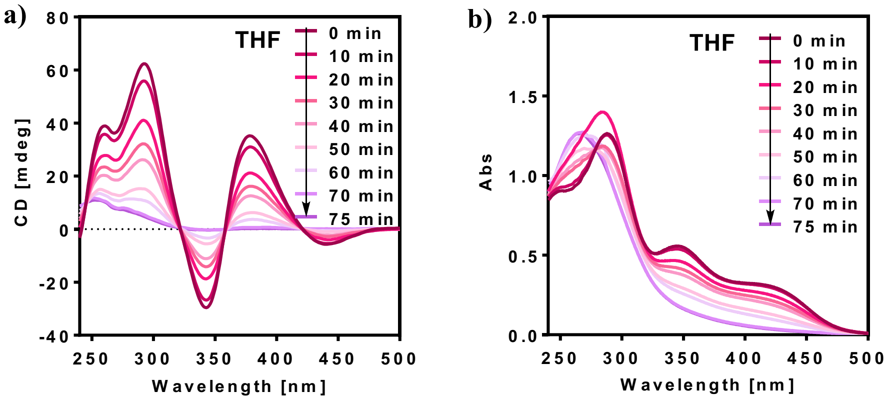

Supplementary Figure 52. (a) CD and (b) UV spectra of poly[(*R*)-**2**$_{0.7}$-co-(*S*)-**2**$_{0.3}$] in THF after irradiation under visible light. [poly[(*R*)-**2**$_{0.7}$-co-(*S*)-**2**$_{0.3}$]]= 9.00·10^{-4} M.

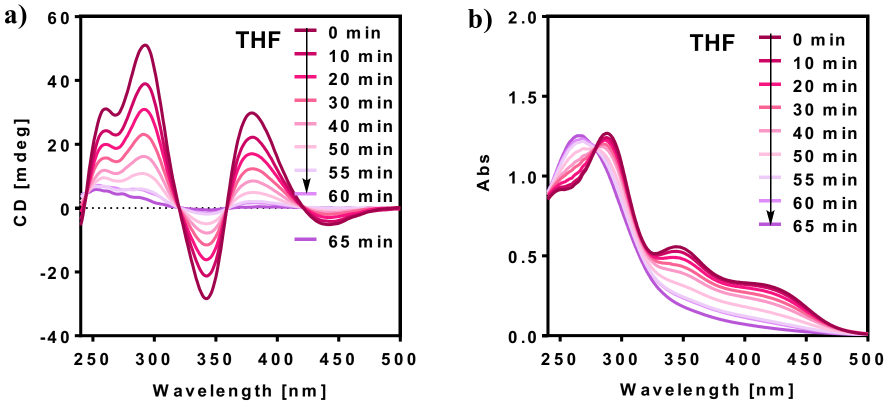

Supplementary Figure 53. (a) CD and (b) UV spectra of poly[(*R*)-**2**$_{0.6}$-co-(*S*)-**2**$_{0.4}$] in THF after irradiation under visible light. [poly[(*R*)-**2**$_{0.6}$-co-(*S*)-**2**$_{0.4}$]]= 9.00·10^{-4} M.

Poly[(*R*)-**8**$_{r}$-co-(*S*)-**8**$_{(1-r)}$] series

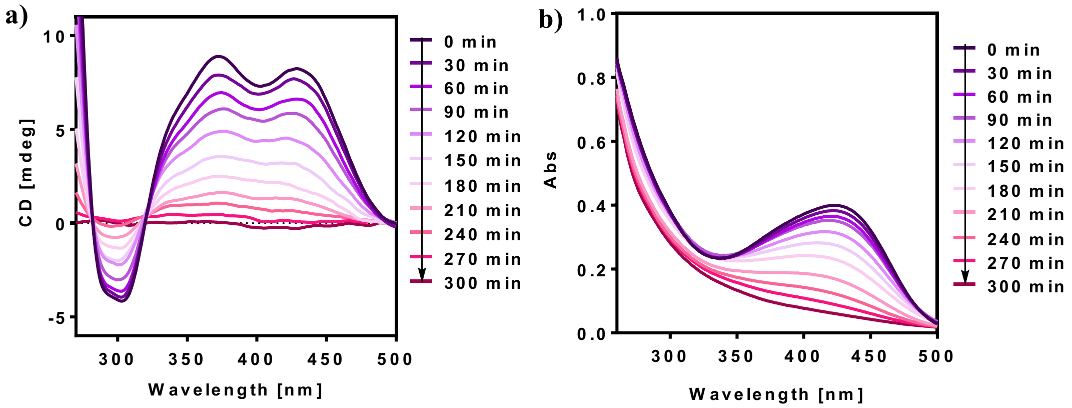

Supplementary Figure 54. (a) CD and (b) UV spectra of poly[(*R*)-**2**$_{0.9}$-co-(*S*)-**2**$_{0.1}$] in $CHCl_3$ after irradiation under visible light. [poly[(*R*)-**8**$_{0.9}$-co-(*S*)-**8**$_{0.1}$]]= 1.02·10^{-3} M.

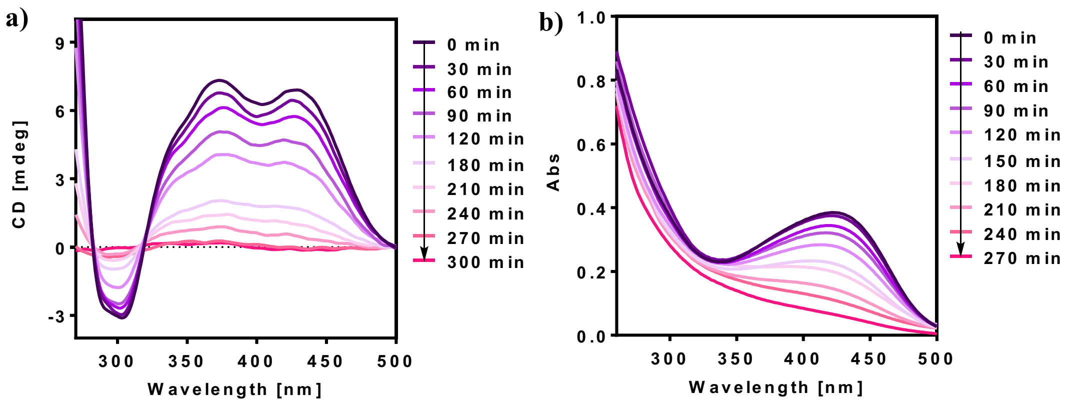

Supplementary Figure 55. (a) CD and (b) UV spectra of poly[(*R*)-**2**$_{0.8}$-co-(*S*)-**2**$_{0.2}$] in $CHCl_3$ after irradiation under visible light. [poly[(*R*)-**8**$_{0.8}$-co-(*S*)-**8**$_{0.2}$]]= $1.02 \cdot 10^{-3}$ M.

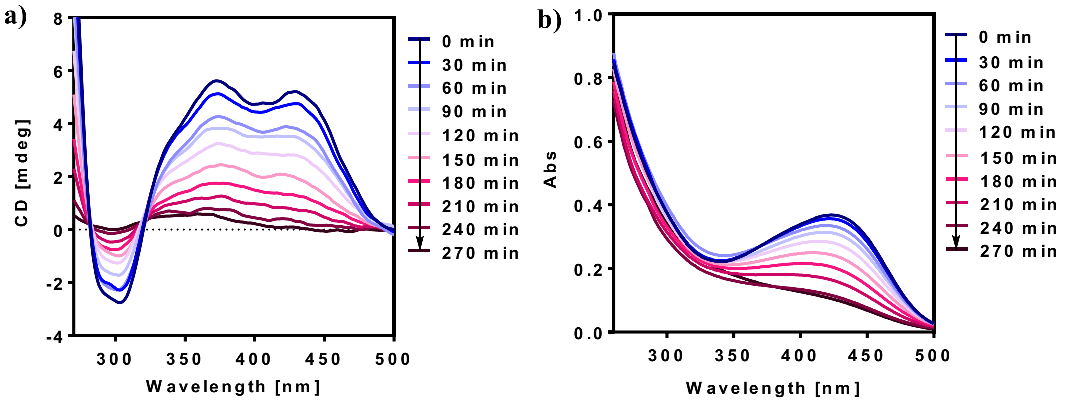

Supplementary Figure 56. (a) CD and (b) UV spectra of poly[(*R*)-**2**$_{0.7}$-co-(*S*)-**2**$_{0.3}$] in $CHCl_3$ after irradiation under visible light. [poly[(*R*)-**8**$_{0.7}$-co-(*S*)-**8**$_{0.3}$]]= $1.02 \cdot 10^{-3}$ M.

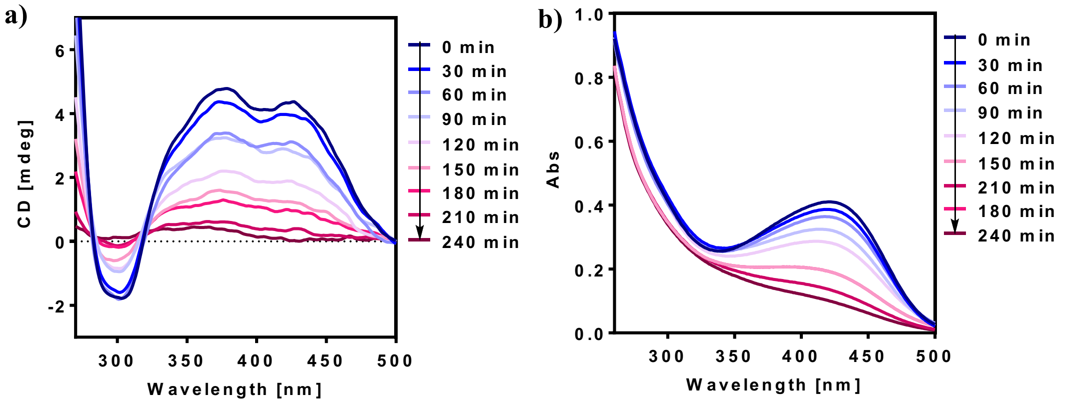

Supplementary Figure 57. (a) CD and (b) UV spectra of poly[(*R*)-**2**$_{0.6}$-co-(*S*)-**2**$_{0.4}$] in $CHCl_3$ after irradiation under visible light. [poly[(*R*)-**8**$_{0.6}$-co-(*S*)-**8**$_{0.4}$]]= $1.02 \cdot 10^{-3}$ M.

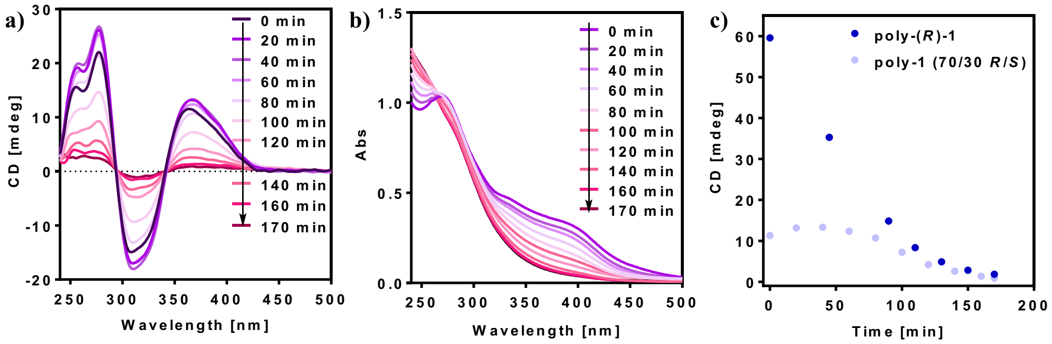

Supplementary Figure 58. (a) CD and (b) UV spectra of a mixture of poly-(*R*)-**1** and poly-(*S*)-**1** in a 7/3 relation in $CHCl_3$ after irradiation under visible light. (c) CD signal decay vs. time for poly-(*R*)-1 and a mixture of poly-(*R*)-**1** and poly-(*S*)-**1** in a 7/3 relation at different irradiation times under visible light in $CHCl_3$ (CD@368nm). [poly-**1**]= $1.02 \cdot 10^{-3}$ M.

Supplementary Tables

Supplementary Table 1. Polymerization conditions of the corresponding homopolymers.

Polymer	Mass (mg)	THF (mL)	$[Rh(nbd)Cl]_2$ (mg)	NEt_3 (μL)	Yield (%)
Poly-(*S*)-**1**	75	1.0	1.0	5	89
Poly-(*R*)-**1**	75	1.0	1.0	5	90
Poly-(S)-**2**	75	1.0	1.0	5	88
Poly-(S)-**2**	75	1.0	1.0	5	87
Poly-(S)-**3**	75	1.0	1.0	5	84
Poly-(*R*)-**4**	75	1.0	1.0	5	92
Poly-(*S*)-**5**	75	1.0	1.0	5	88
Poly-(*S*)-**6**	75	1.0	1.0	5	88
Poly-(*S*)-**7**	75	1.0	1.0	5	87
Poly-(*S*)-**8**	75	1.0	1.0	5	86
Poly-(*R*)-**8**	75	1.0	1.0	5	87

Supplementary Table 2. Polymerization conditions for poly[(*R*)-**1**$_r$-co-(*S*)-**1**$_{(1-r)}$] series.

Copolymer	M-(*R*)-**1** (mg)	M-(*R*)-**1** (mmol)	M-(*S*)-**1** (mg)	M-(*S*)-**1** (mmol)	[Rh(nbd)Cl]$_2$ (mg)	NEt$_3$ (μL)	Yield (%)
poly[(*R*)-**1**$_{0.95}$-co-(*S*)-**1**$_{0.05}$]	83.6	0.285	4.4	0.015	1.3	5	90
poly[(*R*)-**1**$_{0.9}$-co-(*S*)-**1**$_{0.1}$]	79.2	0.270	8.8	0.030	1.3	5	88
poly[(*R*)-**1**$_{0.8}$-co-(*S*)-**1**$_{0.2}$]	74.8	0.255	13.2	0.045	1.3	5	89
poly[(*R*)-**1**$_{0.7}$-co-(*S*)-**1**$_{0.3}$]	70.4	0.240	17.6	0.060	1.3	5	89
poly[(*R*)-**1**$_{0.6}$-co-(*S*)-**1**$_{0.4}$]	52.8	0.180	35.2	0.120	1.3	5	87

Supplementary Table 3. Polymerization conditions for poly[(*R*)-**2**$_r$-co-(*S*)-**2**$_{(1-r)}$] series.

Copolymer	M-(*R*)-**2** (mg)	M-(*R*)-**2** (mmol)	M-(*S*)-**2** (mg)	M-(*S*)-**2** (mmol)	[Rh(nbd)Cl]$_2$ (mg)	NEt$_3$ (μL)	Yield (%)
poly[(*R*)-**2**$_{0.9}$-co-(*S*)-**2**$_{0.1}$]	90.0	0.270	10.0	0.030	1.3	5	91
poly[(*R*)-**2**$_{0.8}$-co-(*S*)-**2**$_{0.2}$]	80.0	0.240	20.0	0.060	1.3	5	90
poly[(*R*)-**2**$_{0.7}$-co-(*S*)-**2**$_{0.3}$]	70.0	0.210	30.0	0.090	1.3	5	88
poly[(*R*)-**2**$_{0.6}$-co-(*S*)-**2**$_{0.4}$]	60.0	0.180	40.0	0.120	1.3	5	88

Supplementary Table 4. Polymerization conditions for poly[(*R*)-**8**$_r$-co-(*S*)-**8**$_{(1-r)}$] series.

Copolymer	M-(*R*)-**8** (mg)	M-(*R*)-**8** (mmol)	M-(*S*)-**8** (mg)	M-(*S*)-**8** (mmol)	[Rh(nbd)Cl]$_2$ (mg)	NEt$_3$ (μL)	Yield (%)
poly[(*R*)-**8**$_{0.9}$-co-(*S*)-**8**$_{0.1}$]	79.2	0.270	8.8	0.030	1.3	5	85
poly[(*R*)-**8**$_{0.8}$-co-(*S*)-**8**$_{0.2}$]	74.8	0.255	13.2	0.045	1.3	5	84
poly[(*R*)-**8**$_{0.7}$-co-(*S*)-**8**$_{0.3}$]	70.4	0.240	17.6	0.060	1.3	5	84
poly[(*R*)-**8**$_{0.6}$-co-(*S*)-**8**$_{0.4}$]	52.8	0.180	35.2	0.120	1.3	5	82

Supplementary Table 5. Original data from ECD signal of irradiation experiments to calculate equations 3-6.

% comonomer	Equation 3		Equation 4		Equation 5		Equation 6
	**t_{50}**	**t_{20}**	**t_{50}**	**t_{20}**	**t_{50}**	**t_{20}**	**t_{20}**
**0**	55	95	61	97	21	35	220
**5**	43	70					
**10**	41	64	55	80	16	24	202
**20**	21	33	40	63	11	21	197
**30**	13	23	27	48	10	19	190
**40**	10	19	24	26	8	16	184

Supplementary Table 6. GPC data of poly-(*R*)-**1** and poly[(*R*)-$\mathbf{1}_{r}$-co-(*S*)-$\mathbf{1}_{(1-r)}$] series.

Polymer	Mn	Mw	Đ
Poly-(*R*)-**2**	24800	35000	1.41
poly[(*R*)-$\mathbf{2}_{0.9}$-co-(*S*)-$\mathbf{2}_{0.1}$]	13500	17000	1.53
poly[(*R*)-$\mathbf{2}_{0.8}$-co-(*S*)-$\mathbf{2}_{0.2}$]	25400	42500	1.67
poly[(*R*)-$\mathbf{2}_{0.7}$-co-(*S*)-$\mathbf{2}_{0.3}$]	20600	36900	1.79
poly[(*R*)-$\mathbf{2}_{0.6}$-co-(*S*)-$\mathbf{2}_{0.4}$]	24500	40900	1.67

Supplementary Table 7. GPC data of poly-(*R*)-**2** and poly[(*R*)-$\mathbf{2}_{r}$-co-(*S*)-$\mathbf{2}_{(1-r)}$] series.

Polymer	Mn	Mw	Đ
poly-(*R*)-**1**	12400	19500	1.57
poly[(*R*)-$\mathbf{1}_{0.95}$-co-(*S*)-$\mathbf{1}_{0.05}$]	5600	7100	1.26
poly[(*R*)-$\mathbf{1}_{0.9}$-co-(*S*)-$\mathbf{1}_{0.1}$]	13000	15600	1.49
poly[(*R*)-$\mathbf{1}_{0.8}$-co-(*S*)-$\mathbf{1}_{0.2}$]	7900	11800	1.49
poly[(*R*)-$\mathbf{1}_{0.7}$-co-(*S*)-$\mathbf{1}_{0.3}$]	10700	13200	1.24
poly[(*R*)-$\mathbf{1}_{0.6}$-co-(*S*)-$\mathbf{1}_{0.4}$]	25400	32900	1.57

Supplementary Table 8. GPC data of poly-(*R*)-**8** and poly[(*R*)-$\mathbf{8}_{r}$-co-(*S*)-$\mathbf{8}_{(1-r)}$] series.

Polymer	Mn	Mw	Đ
Poly-(*R*)-**8**	37300	51100	1.37
poly[(*R*)-$\mathbf{8}_{0.9}$-co-(*S*)-$\mathbf{8}_{0.1}$]	33900	50300	1.48
poly[(*R*)-$\mathbf{8}_{0.8}$-co-(*S*)-$\mathbf{8}_{0.2}$]	31500	44000	1.39
poly[(*R*)-$\mathbf{8}_{0.7}$-co-(*S*)-$\mathbf{8}_{0.3}$]	39100	52800	1.35
poly[(*R*)-$\mathbf{8}_{0.6}$-co-(*S*)-$\mathbf{8}_{0.4}$]	25200	34900	1.38

Supplementary Table 9. GPC data of poly-(*S*)-**3**, poly-(*R*)-**4**, poly-(*S*)-**5**, poly-(*S*)-**6** and poly-(*S*)-**7**.

Polymer	Mn	Mw	Đ
Poly-(*S*)-**3**	32300	48000	1.49
Poly-(*R*)-**4**	10200	16100	1.58
Poly-(*S*)-**5**	21000	34600	1.65
Poly-(*S*)-**6**	24300	41300	1.70
Poly-(*S*)-**7**	19500	32800	1.68

Supplementary References

1. I. Louzao, J. M. Seco, E. Quiñoá, R. Riguera, *Angew. Chem. Int. Ed.* **2010**, *49*, 1430-1433.

2. S. Leiras, F. Freire, J. M. Seco, E. Quiñoá, R. Riguera, *Chem. Sci.* **2013**, *4*, 2735-2743.

3. K. Cobos, R. Rodríguez, E. Quiñoá, R. Riguera, F. Freire, *Angew. Chem. Int. Ed.* **2020**, *59*, 23724-23730.

4. S. Arias, M. Núñez-Martínez, E. Quiñoá, R. Riguera, F. Freire, *Polym. Chem.* **2017**, *8*, 3740-3745.

5. S. Arias, R. Rodríguez, E. Quiñoá, R. Riguera, F. Freire, *J. Am. Chem. Soc.* **2018**, *140*, 667-674.

6. M. Alzubi, S. Arias, I. Louzao, E. Quiñoá, R. Riguera, F. Freire, *Chem. Commun*. **2017**, *53*, 8573-8576.

7. R. Rodríguez, E. Quiñoá, R. Riguera, F. Freire, *J. Am. Chem. Soc.* **2016**, *138*, 9620-9628.

8. F. Rey-Tarrío, R. Rodríguez, E. Quiñoá, R. Riguera, F. Freire, *Angew. Chem. Int. Ed.* **2021**, *60*, 8095-8103.
